# Fam64a is a novel cell cycle promoter of hypoxic fetal cardiomyocytes in mice

**DOI:** 10.1038/s41598-017-04823-1

**Published:** 2017-06-30

**Authors:** Ken Hashimoto, Aya Kodama, Takeshi Honda, Akira Hanashima, Yoshihiro Ujihara, Takashi Murayama, Shin-ichiro Nishimatsu, Satoshi Mohri

**Affiliations:** 1 First Department of Physiology, Kawasaki Medical School, Kurashiki, Okayama, 701-0192 Japan; 2 Department of Cardiovascular Surgery, Kawasaki Medical School, Kurashiki, Okayama, 701-0192 Japan; 3 Department of Cellular and Molecular Pharmacology, Juntendo University, Tokyo, 113-8421 Japan; 4 Department of Molecular and Developmental Biology, Kawasaki Medical School, Kurashiki, Okayama, 701-0192 Japan

## Abstract

Fetal cardiomyocytes actively proliferate to form the primitive heart in utero in mammals, but they stop dividing shortly after birth. The identification of essential molecules maintaining this active cardiomyocyte proliferation is indispensable for potential adult heart regeneration. A recent study has shown that this proliferation depends on a low fetal oxygen condition before the onset of breathing at birth. We have established an isolation protocol for mouse fetal cardiomyocytes, performed under strict low oxygen conditions to mimic the intrauterine environment, that gives the highest proliferative activities thus far reported. Oxygen exposure during isolation/culture markedly inhibited cell division and repressed cell cycle-promoting genes, and subsequent genome-wide analysis identified Fam64a as a novel regulatory molecule. Fam64a was abundantly expressed in hypoxic fetal cardiomyocyte nuclei, but this expression was drastically repressed by oxygen exposure, and in postnatal cardiomyocytes following the onset of breathing and the resulting elevation of oxygen tension. Fam64a knockdown inhibited and its overexpression enhanced cardiomyocyte proliferation. Expression of a non-degradable Fam64a mutant suggested that optimum Fam64a expression and subsequent degradation by anaphase-promoting complex/cyclosome (APC/C) during the metaphase-to-anaphase transition are required for fetal cardiomyocyte division. We propose that Fam64a is a novel cell cycle promoter of hypoxic fetal cardiomyocytes in mice.

## Introduction

Fetal cardiomyocytes (fCMs) actively proliferate in utero in mammals to form the primitive heart, but they stop dividing soon after birth and switch to hypertrophic growth. Understanding the molecular mechanisms of active fCM proliferation is fundamental for potential therapeutic regeneration in adult hearts. Recently, Puente *et al*. have shown that a low oxygen (O_2_) condition in fetal hearts, prior to the onset of breathing at birth, is crucial for the active proliferation of fCMs^1^. However, the molecular mechanism underlying this induction of fCM proliferation by low O_2_ conditions is unknown. One recent study has shown that proliferation of mouse fCMs during mid-embryonic stage (embryonic day, E12.5–E14.5) was maintained by hypoxia inducible factor-1α (Hif-1α), a key molecule in the hypoxic response, along with various other molecules, including macrophage migration inhibitory factor (Mif) and p53^2^. Hif-1α showed a characteristic accumulation in the nucleus, which was correlated with active fCM proliferation specifically within this stage. However, at the later embryonic stages beyond E14.5, Hif-1α was no longer observed in the nucleus^2^, and the nature of other essential factors required at this stage and until birth remains unclear.

The fCMs are recognized as actively proliferating cells in utero^3, 4^, but for as yet unknown reasons, the current standard cell culture protocols do not reproduce this active proliferation, which prevents mechanistic cell cycle analysis of these cells. Consequently, most studies evaluate proliferation using indirect methods, such as analyses of DNA synthesis or cell cycle marker expression. However, these analyses cannot correctly evaluate cell division, especially for CMs, as these cells sometimes undergo karyokinesis without cytokinesis and become multinucleate^3^. Thus, the inability to observe CM cell division directly, and with a sufficiently frequent occurrence for mechanistic analysis, is a crucial limitation. One approach has been to use time-lapse analyses of heart slice cultures^5^ and mosaic analyses with double markers for heart tissues^6^, but no quantitative evaluation at a single-cell level has yet been described. Studies that include time-lapse imaging in neonatal^7^ and adult^8^ CMs have revealed only a limited occurrence of cytokinesis, with a rate of only ~0.6% reported in the adult cell study.

The observation that active fCM proliferation *in vivo* appears to require a low O_2_ condition^1^ prompted us to hypothesize that the exposure of CMs to ambient air (21% O_2_), as would occur when using conventional isolation protocols, would suppress the proliferative ability of the resulting isolated fCMs. Therefore we established an isolation protocol that could be performed under strict low O_2_ conditions that would mimic the in utero environment (reportedly 20–25 mmHg, or 2.6–3.2% O_2_)^9^. Our new protocol increased the rate of completed division to ~5% in mouse fCMs, which was clearly observable using time-lapse imaging. To the best of our knowledge, this cell division rate is the highest yet reported for mouse CMs.

We cultured late embryonic fCMs (E16–E18) using this culture system and were able to identify Fam64a (family with sequence similarity 64, member A, also known as Rcs1) as the essential molecule for fCM proliferation. Fam64a is recognized as a cell cycle promoter localized in the nucleus of rapidly proliferating cells (e.g., HeLa cells)^10–12^, but it has no known function in CMs. In HeLa cells, Fam64a is degraded by the anaphase-promoting complex/cyclosome (APC/C) at mitotic exit, and appears to control the timing of the metaphase-to-anaphase transition by interacting with chromatin remodelers^11^. The APC/C plays a central role in sister chromatid segregation during the metaphase-to-anaphase transition, where it serves as a component of ubiquitin-proteasome system^13–15^. However, in HeLa cells, alteration of Fam64a expression by knockdown, overexpression, or non-degradable mutant expression has no effect on cell proliferation, and mitosis proceeds normally^11^, which suggests that Fam64a is not essential for proliferation of these cells. Here, we report that Fam64a is indispensable for fCM proliferation, where its optimum expression and degradation by the APC/C are both required for the cell cycle to progress. This degradation occurs during the metaphase-to-anaphase transition, which is an earlier time point than is seen in HeLa cells. Fam64a expression is induced under low O_2_ conditions, independently of Hif-1α. Our findings identify a novel O_2_-dependent and Hif-1α-independent system that is essential for fCM proliferation at the late embryonic stage.

## Results

### Exposure of fCMs to O_2_ inhibits proliferation and cell cycle activity

**Figure 1:**
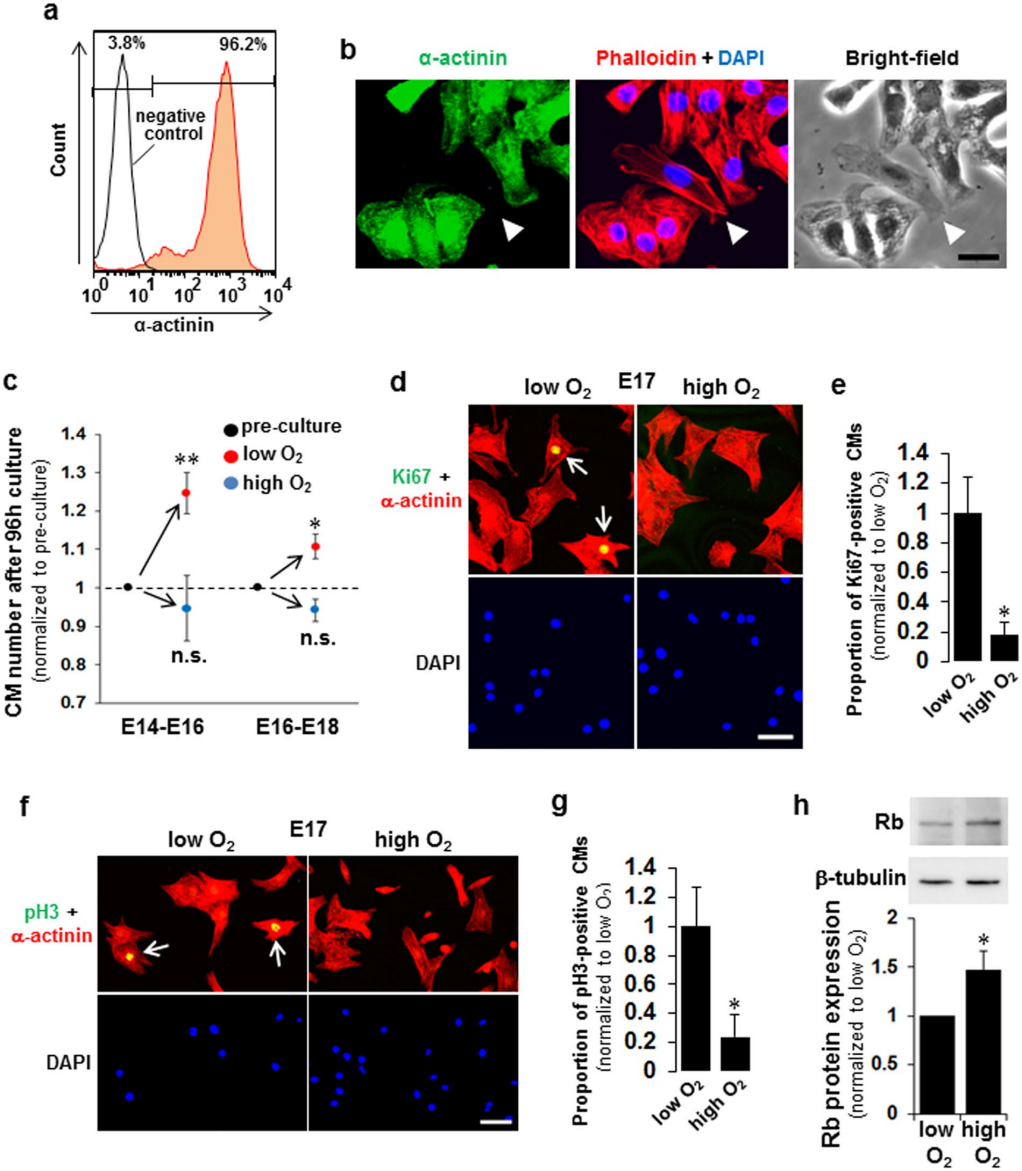
Exposure of fetal cardiomyocytes (fCMs) to O_2_ inhibits proliferation and cell cycle activity. (**a**) FACS analyses of E16 fCMs stained for α-actinin indicate > 95% purity when isolated and cultured under low O_2_ conditions. (**b**) Immunofluorescence for α-actinin observed in phalloidin, DAPI, and bright-field images of E16 fCMs. The arrowhead indicates one of the few contaminating fibroblasts, which have a distinct phase appearance and are completely negative for α-actinin. (**c**) The fCMs (E14–E16 or E16–E18) isolated under low O_2_ conditions were cultured under low or high O_2_ conditions for 96 h. At the start and end of the culture, total cell numbers were counted, and the proportion of CMs and non-CMs were determined by FACS to obtain the absolute number of each cell type. n = 3–5 independent experiments. ^*^ *P* < 0.05 and ^**^ *P* < 0.01 compared to pre-culture levels. n.s. = not significant. (**d**,**f**) Immunofluorescence for Ki67 (**d**) and pH3 (**f**) observed in α-actinin and DAPI of low O_2_-isolated fCMs cultured under low or high O_2_ conditions. Arrows denote Ki67- (**d**) and pH3- (**f**) positive fCMs. (**e**,**g**) Proportions of Ki67- (**e**) and pH3- (**g**) positive CMs in (**d**) and (**f**), respectively. n = 5 independent experiments. ^*^ *P* < 0.05. (**h**) Rb protein expressions in low O_2_-isolated fCMs (E16–E17) cultured under low or high O_2_ conditions. β-tubulin was used as a loading control. n = 5 independent experiments. ^*^ *P* < 0.05. Error bars = SEM. Scale bars = 20 µm in (**b**) and 50 µm in (**d**,**f**).

In this study, we used fCMs mainly at the E16–E18 stage to elucidate the molecular mechanism of fCM proliferation at the late embryonic stage. Our protocol established for cell isolation under low O_2_ conditions consistently yielded healthy fCMs with > 95% purity and few contaminating non-CMs (mostly fibroblasts). The purity was evaluated by FACS using sarcomeric α-actinin as a specific CM marker (Fig. 1a). Fibroblasts have a distinct phase contrast appearance and are completely negative for α-actinin staining (Fig. 1b). The fCMs isolated under low O_2_ conditions were then cultured under low O_2_ (2–3%) or high O_2_ (21%) conditions for 96 h. The CM numbers increased 1.1–1.3-fold under low O_2_ conditions, but no increase was observed under high O_2_ conditions, indicating a significant inhibition of proliferation following exposure of fCMs to O_2_ (Fig. 1c). Exposure to O_2_ also significantly decreased the proportion of fCMs showing positivity for Ki67, a cell cycle marker, and phospho-histone H3 (pH3), a mitosis marker (Fig. 1d–g). The expression of retinoblastoma protein (Rb), a negative cell cycle regulator, was also elevated following O_2_ exposure (Fig. 1h). A DNA microarray also showed that O_2_ exposure drastically decreased the activity of cell cycle-promoting gene pathways (the top two downregulated pathways) (Table 1). These data indicate that O_2_ exposure inhibits proliferation and cell cycle activity of late embryonic stage fCMs.

**Table 1:** Top 10 up or downregulated pathways in microarray #2 (high O_2_ compared to low O_2_).

	MAPP name	Z score	P value
**Upregulated (ratio > 1.5)**			
1	Mm_Kit_Receptor_Signaling_Pathway_WP407_35754	4.15	0.001
2	Mm_Myometrial_Relaxation_and_Contraction_Pathways_WP385_35258	3.01	0.005
3	Mm_Apoptosis_WP1254_35103	3.23	0.006
4	Mm_Apoptosis_Mechanisms_WP168_34410	3.19	0.006
5	Mm_Striated_Muscle_Contraction_WP216_33380	2.98	0.011
6	Mm_T_Cell_Receptor_Signaling_Pathway_WP480_34406	2.54	0.018
7	Mm_IL-5_Signaling_Pathway_WP151_34416	2.42	0.03
8	Mm_Calcium_Regulation_in_the_Cardiac_Cell_WP553_35443	2.12	0.038
9	Mm_Apoptosis_Modulation_by_HSP70_WP166_32716	2.25	0.078
10	Mm_Oxidative_Damage	2.03	0.083
**Downregulated (ratio < 0.67)**			
1	Mm_DNA_Replication_WP150_35196	10.23	0
2	Mm_Cell_cycle_WP190_35363	7.67	0
3	Mm_Glycolysis_and_Gluconeogenesis_WP157_34413	7.07	0
4	Mm_Glycolysis_Gluconeogenesis	6.47	0
5	Mm_Striated_Muscle_Contraction_WP216_33380	6.12	0
6	Mm_G1_to_S_cell_cycle_control_WP413_35821	5.64	0
7	Mm_Fructose_and_mannose_metabolism	3.41	0.009
8	Mm_Electron_Transport_Chain_WP295_35239	2.73	0.009
9	Mm_Galactose_metabolism	3.53	0.013
10	Mm_Pentose_phosphate_pathway	3.05	0.019

MAPP = Map Annotator and Pathway Profiler.

### Direct observation reveals O_2_-induced inhibition of fCM cell division

**Figure 2:**
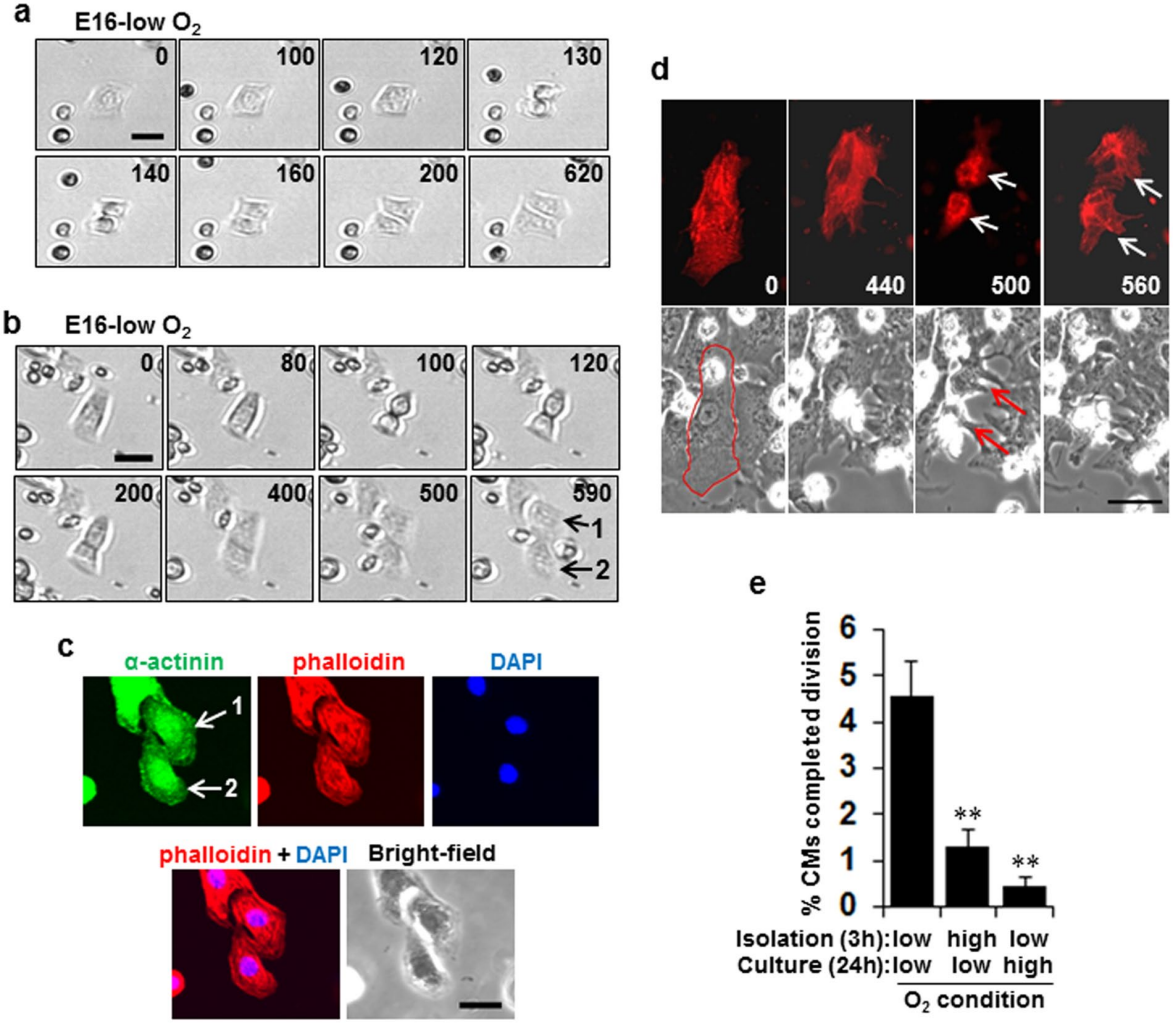
Direct observation reveals O_2_-induced inhibition of fetal cardiomyocyte (fCM) cell division. (**a,b**) Two representative time-lapse recordings of cell division dynamics of E16 fCMs under low O_2_ isolation and culture conditions. The number on each panel indicates the time (in minutes) elapsed from the time indicated in the first panel. (**c**) Post-imaging sample of (**b**) containing two daughter cells following cell division (numbered as 1 and 2 on the last panel of (**b**)). The sample was fixed and immunostained for α-actinin and observed in phalloidin, DAPI, and bright-field images. Two identical daughter cells were then imaged (numbered as 1 and 2 in the upper left panel of (**c**)). This correlative analysis confirmed that the dividing cells were unequivocally CMs. (**d**) A representative time-lapse recording of cell division dynamics of fCMs transduced with sarcomeric α-actinin-mCherry baculovirus observed in bright-field images. The number on each panel indicates the time (in minutes) elapsed from the time indicated in the first panel. The event of mitosis and cytokinesis was marked by arrows. The contour of the fCM at time 0 was outlined in red. (**e**) Percentage of E16 fCMs that completed cell division as determined by time-lapse imaging under the indicated conditions. n = 3–6 independent experiments and ~1700 cells were counted for each condition. ^**^ *P* < 0.01 compared to low O_2_ isolation and low O_2_ culturing conditions. Error bars = SEM. Scale bars = 30 µm in (**a**,**b** and **d**) and 20 µm in (**c**).

We used direct time-lapse observation to determine whether exposure of fCMs to O_2_ inhibits the cell division process itself. Figure 2a–b shows two representative recordings of cell division dynamics of E16 fCMs isolated and maintained under low O_2_ conditions. Cytokinesis and the formation of new daughter cells are clearly visible. A movie of the entire time series with additional examples is available in Supplementary Movies 1 and 2. The post-imaging samples that contained two daughter cells from cell division were also fixed and immunostained for sarcomeric α-actinin, and the two identical daughter cells were imaged. This analysis confirmed that the dividing cells were unequivocally CMs (Fig. 2b,c). The identity of the dividing cells was further confirmed by a novel baculovirus-mediated gene transfer^16^, which identified α-actinin-expressing fCMs undergoing cell division (Fig. 2d and Supplementary Movie 3). This time-lapse analysis gave a rate of completed cell division of ~5% (Fig. 2e), which is the highest thus far reported for mouse CMs. Notably, even short-term ambient O_2_ exposure during isolation (~3 h) markedly inhibited fCM division by 72%, and longer exposure during culture (~24 h) further inhibited division by 90% (Fig. 2e). These observations confirmed the absolute requirement for strict maintenance of low O_2_ conditions for fCM proliferation to proceed.

The time-lapse analyses revealed that nearly all the dividing CMs were mononucleate, and they generated two daughter cells that were also mononucleate, suggesting that the daughter cells were also still potentially cycling after division. Prior to cell division, the cells assumed a rounded shape, as is common with proliferating cells^17^ (Fig. 2a–b and Supplementary Movies 1 and 2). Estimates made based on cell shape changes indicated that the transition from anaphase to telophase lasted ~15 min and cytokinesis lasted ~35 min. These durations were unaffected by O_2_ conditions (Supplementary Fig. 1a,b).

### Fam64a is identified as an essential molecule for fCM proliferation at the late embryonic stage

The critical molecule responsible for the induction of fCM proliferation by low O_2_ conditions was investigated by first checking for any involvement of p53, the well-known master regulator for cell cycle arrest. The p53 levels were not altered either by exposure to O_2_ in fCMs or in heart tissues at birth (Supplementary Fig. 2a,b). Similarly, no changes in level were noted at birth for the mammalian target of rapamycin (mTOR), a cell growth master regulator, or for AMP-activated protein kinase α (AMPKα), including its phosphorylated form, a master regulator of metabolic control (Supplementary Fig. 2c–f).

**Figure 3:**
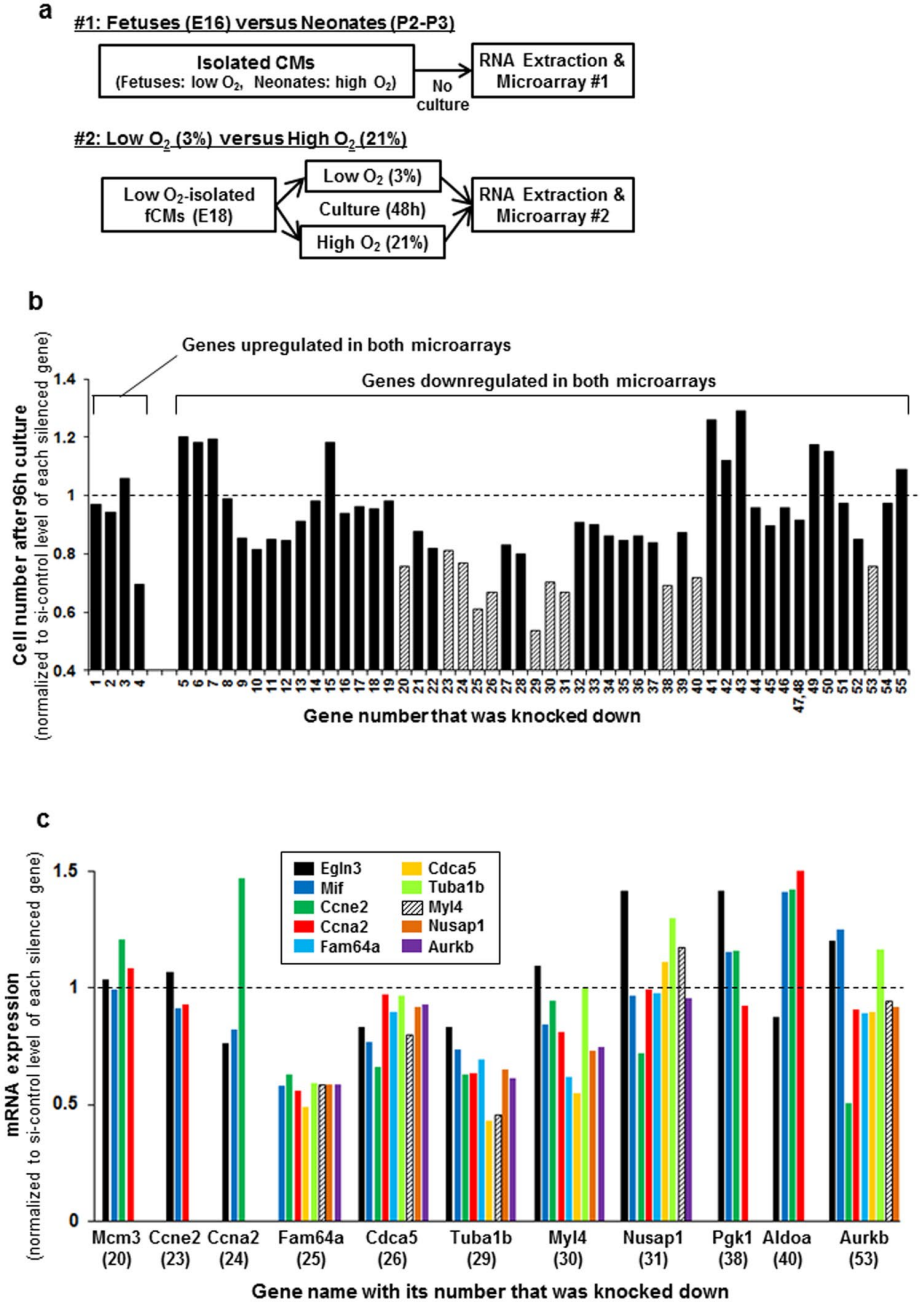
Fam64a is identified as an essential molecule for fetal cardiomyocyte (fCM) proliferation at the late embryonic stage. (**a**) DNA microarrays were performed with two sample sets—array #1: fetuses versus neonates, and array #2: fCMs cultured under low versus high O_2_ conditions. For array #1, CMs were isolated from fetal hearts under low O_2_ conditions or from neonatal hearts under high O_2_ conditions, and total RNA was immediately obtained (with no further cell culturing). In array #2, fCMs isolated under low O_2_ conditions were separately cultured under low or high O_2_ conditions, and then total RNA was extracted. (**b**) Each of 55 selected genes was knocked down in late embryonic stage fCMs (E16–E17) isolated under low O_2_ conditions. The cells were cultured for 96 h, and proliferative activity was evaluated by cell counting. Eleven genes (hatched bars) were selected as showing strong proliferation-inhibiting effects when knocked down. The *Hist1h2ao* (No. 47) and *Hist1h2af* (No. 48) genes had no specific siRNA available; therefore, an siRNA targeting both genes was used. Data are shown as normalized to the si-control level of each silenced gene, set at 1. This experiment was performed once for screening purposes. (**c**) In fCMs (E16–E17) isolated under low O_2_ conditions, each of the 11 genes selected in (**b**) was knocked down and the mRNA levels of the indicated genes were evaluated by qPCR. Knockdown of Fam64a resulted in the strongest repression of cell cycle-promoting genes. Data are shown as normalized to the si-control level of each silenced gene, set at 1. This experiment was performed once for screening purposes.

Genome-wide screening by DNA microarray on fetuses versus neonates (#1) and fCMs cultured under low versus high O_2_ conditions (#2) (Fig. 3a) revealed complicated changes occurring at birth, whereas the results for the fCMs in array #2 allowed segregation of the effects of O_2_ changes. The genes showing common expression profiles in both arrays were considered critical genes. The reliability of the microarray analysis was verified by quantitative PCR (qPCR) (Supplementary Fig. 3a,b). Of ~23,500 mouse genes, 640 genes were upregulated and 1,124 were downregulated in array #1. In array #2, 89 genes were upregulated and 136 were downregulated (all the data can be found in Supplementary Dataset 1 and 2). Pathway analyses clearly indicated a drastic repression of cell cycle-promoting gene pathways both by O_2_ exposure (array #2; the top two downregulated pathways) (Table 1) and after birth (array #1; the top three downregulated pathways) (Table 2), confirming that a low O_2_ condition is indispensable for active cell cycle progression in fCMs. We also noted a metabolic shift in the CMs from anaerobic glycolysis to aerobic mitochondrial fatty acid β-oxidation after birth (Table 2)^1, 18^ and following O_2_ exposure (Table 1), suggesting that the high cell cycle activity of fCMs is associated with low O_2_-consuming energetics before birth.

**Table 2:** Top 10 up or downregulated pathways in microarray #1 (neonates compared to fetuses).

	MAPP name	Z score	P value
**Upregulated (ratio > 2.0)**			
1	Mm_Fatty_acid_metabolism	5.34	0
2	Mm_Tryptophan_metabolism	4.68	0
3	Mm_Fatty_Acid_Beta_Oxidation_WP1269_34394	4.42	0
4	Mm_Triacylglyceride_Synthesis_WP386_21490	4.79	0.001
5	Mm_Fatty_Acid_Beta_Oxidation_1_WP221_21143	4.23	0.001
6	Mm_Bile_acid_biosynthesis	4.23	0.004
7	Mm_Mitochondrial_LC-Fatty_Acid_Beta-Oxidation_WP401_34420	4.25	0.005
8	Mm_Butanoate_metabolism	3.89	0.006
9	Mm_Complement_and_Coagulation_Cascades_KEGG_WP449_33690	3.25	0.006
10	Mm_gamma_Hexachlorocyclohexane_degradation	3.09	0.012
**Downregulated (ratio < 0.5)**			
1	Mm_Cell_cycle_WP190_35363	8.10	0
2	Mm_DNA_Replication_WP150_35196	7.99	0
3	Mm_G1_to_S_cell_cycle_control_WP413_35821	5.64	0
4	Mm_Glycolysis_and_Gluconeogenesis_WP157_34413	4.23	0.002
5	Mm_One_Carbon_Metabolism_WP435_34970	3.61	0.003
6	Mm_Nucleotide_Metabolism_WP87_33534	3.29	0.005
7	Mm_Starch_and_sucrose_metabolism	2.86	0.008
8	Mm_Pentose_phosphate_pathway	2.97	0.013
9	Mm_Glycolysis_Gluconeogenesis	2.52	0.018
10	Mm_One_carbon_pool_by_folate	2.84	0.024

MAPP = Map Annotator and Pathway Profiler.

We selected 55 critical genes for further analysis whose expression profiles were common in both arrays (Table 3). Of these, four genes (Nos 1–4) were upregulated and 51 genes (Nos 5–55) were downregulated. Cell counting proliferation assays narrowed the candidates to 11 genes that showed a strong proliferation-inhibiting effect when knocked down (Fig. 3b; hatched bars). These genes included *Mcm3* (20), *Ccne2* (23), *Ccna2* (24), *Fam64a* (25), *Cdca5* (26), *Tuba1b* (29), *Myl4* (30), *Nusap1* (31), *Pgk1* (38), *Aldoa* (40), and *Aurkb* (53) (each gene symbol, with its assigned number in parentheses; see Table 3 for details). Subsequent qPCR screening identified *Fam64a* as showing the strongest repression of cell cycle-promoting genes when knocked down (Fig. 3c). The knockdown efficiency (>80%) was verified by qPCR (Supplementary Fig. 3c).

**Table 3:** Critical genes selected from two microarrays.

No	Symbol	ID	Description	Aliases
**Genes upregulated in both microarrays**				
1	Btg2	12227	B-cell translocation gene 2, anti-proliferative	AA959598, APRO1, Pc3, TIS21
2	Ccng1	12450	cyclin G1	AI314029
3	9030617O03Rik	217830	RIKEN cDNA 9030617O03 gene	
4	Myh6	17888	myosin, heavy polypeptide 6, cardiac muscle, alpha	A830009F23Rik, AA517445, Myhc-a, Myhca, alpha-MHC
**Genes downregulated in both microarrays**				
5	Egln3	112407	egl-9 family hypoxia-inducible factor 3	2610021G09Rik, AI505553, AI648162, Hif-p4h-3, Phd3, SM-20
6	Mif	17319	macrophage migration inhibitory factor	GIF, Glif
7	Hif1a	15251	hypoxia inducible factor 1, alpha subunit	AA959795, HIF1alpha, MOP1, bHLHe78
8	Birc5	11799	baculoviral IAP repeat-containing 5	AAC-11, Api4, TIAP, survivin40
9	Ankrd1	107765	ankyrin repeat domain 1 (cardiac muscle)	Alrp, CARP, Crap, MARP1
10	Acta2	11475	actin, alpha 2, smooth muscle, aorta	0610041G09Rik, Actvs, SMalphaA, a-SMA, alphaSMA
11	Rpl41	67945	ribosomal protein L41	1810055P16Rik, 2210411K19Rik
12	Rpl22l1	68028	ribosomal protein L22 like 1	3110001N18Rik, AU041196
13	Slc2a1	20525	solute carrier family 2 (facilitated glucose transporter), member 1	Glut-1, Glut1
14	Slc16a3	80879	solute carrier family 16 (monocarboxylic acid transporters), member 3	Mct3, Mct4
15	Hist1h2ac	319164	histone cluster 1, H2ac	
16	Fam162a	70186	family with sequence similarity 162, member A	2310056P07Rik, HGTD-P
17	Nbeal2	235627	neurobeachin-like 2	1110014F23Rik, BC042396, mKIAA0540
18	Ppia	268373	peptidylprolyl isomerase A	2700098C05, Cphn, CyP-18, CypA
19	Asf1b	66929	anti-silencing function 1B histone chaperone	1700003K02Rik, AA409591
20	Mcm3	17215	minichromosome maintenance deficient 3 (S. cerevisiae)	AL033361, C80350, Mcmd, P1, p1.m
21	Mcm7	17220	minichromosome maintenance deficient 7 (S. cerevisiae)	AI747533, Mcmd7, mCDC47
22	Mcm10	70024	minichromosome maintenance deficient 10 (S. cerevisiae)	2410041F14Rik, AU018508, C330019M07Rik, C79164
23	Ccne2	12448	cyclin E2	
24	Ccna2	12428	cyclin A2	AA408589, Ccn-1, Ccn1, Ccna, CycA2, Cyca
25	Fam64a	109212	family with sequence similarity 64, member A	CATS; AI115087; 2610008F03Rik; 6720460F02Rik
26	Cdca5	67849	cell division cycle associated 5	2610036L13Rik, AL024086, AW536684, C85404
27	Cdk1	12534	cyclin-dependent kinase 1	Cdc2, Cdc2a, p34 <CDC2>
28	Cdc20	107995	cell division cycle 20	2310042N09Rik, C87100, p55CDC
29	Tuba1b	22143	tubulin, alpha 1B	Tuba2
30	Myl4	17896	myosin, light polypeptide 4	ALC1, AMLC, ELC, ELC1a, GT1, MLC1a, Myla
31	Nusap1	108907	nucleolar and spindle / protein 1	2610201A12Rik, AI481307, ANKT, AW547774, BB165529, BM037, LNP, NuSAP, Q0310, SAPL
32	Sash1	70097	SAM and SH3 domain containing 1	1100001C18Rik, 2500002E12Rik, A330076K04Rik, mKIAA0790
33	Mgarp	67749	mitochondria localized glutamic acid rich protein	4930583H14Rik, AI195347, CESP-1, HUMMR, Osap, Qsap
34	Eef1a1	13627	eukaryotic translation elongation factor 1 alpha 1	
35	Pkm	18746	pyruvate kinase, muscle	AA414905, AL024370, AL024424, Pk-2, Pk-3, Pk32, Pkm
36	Pfkfb3	170768	6-phosphofructo-2-kinase/fructose-2,6-biphosphatase 3	E330010H22Rik, iPFK-2, uPFK-2
37	Pdk1	228026	pyruvate dehydrogenase kinase, isoenzyme 1	B830012B01, D530020C15Rik
38	Pgk1	18655	phosphoglycerate kinase 1	Pgk-1
39	Pfkl	18641	phosphofructokinase, liver, B-type	AA407869, ATP-PFK, PFK-L
40	Aldoa	11674	aldolase A, fructose-bisphosphate	Aldo-1, Aldo1
41	Aldoc	11676	aldolase C, fructose-bisphosphate	AI847350, AU040929, Aldo3, Scrg2
42	Pygl	110095	liver glycogen phosphorylase	
43	Ldha	16828	lactate dehydrogenase A	Ldh1, Ldhm, l7R2
44	Pgam1	18648	phosphoglycerate mutase 1	2310050F24Rik, Pgam-1
45	Tpi1	21991	triosephosphate isomerase 1	AI255506, Tpi, Tpi-1
46	Gapdh	14433	glyceraldehyde-3-phosphate dehydrogenase	Gapd
47	Hist1h2ao	665433	histone cluster 1, H2ao	
48	Hist1h2af	319173	histone cluster 1, H2af	H2a-221
49	Hist2h2ac	319176	histone cluster 2, H2ac	H2a-613b
50	Hist1h2ak	319169	histone cluster 1, H2ak	
51	Gm5069	277333	predicted pseudogene 5069	EG277333
52	Aurka	20878	aurora kinase A	AIRK1, ARK-1, AU019385, AW539821, Ark1, Ayk1, Aurora-A, IAK, IAK1, Stk6
53	Aurkb	20877	aurora kinase B	AIM-1, AIRK2, AL022959, Aik2, Aim1, Ark2, AurB, IPL1, STK-1, Stk12, Stk5
54	Acta1	11459	actin, alpha 1, skeletal muscle	AA959943, Acta-2, Acts, Actsk-1
55	Mycn	18109	v-myc myelocytomatosis viral related oncogene, neuroblastoma derived (avian)	N-myc, Nmyc, Nmyc-1, Nmyc1, bHLHe37, c-nmyc

### Abundant nuclear Fam64a expression in hypoxic fCMs is repressed by O_2_ exposure and in postnatal CMs

**Figure 4:**
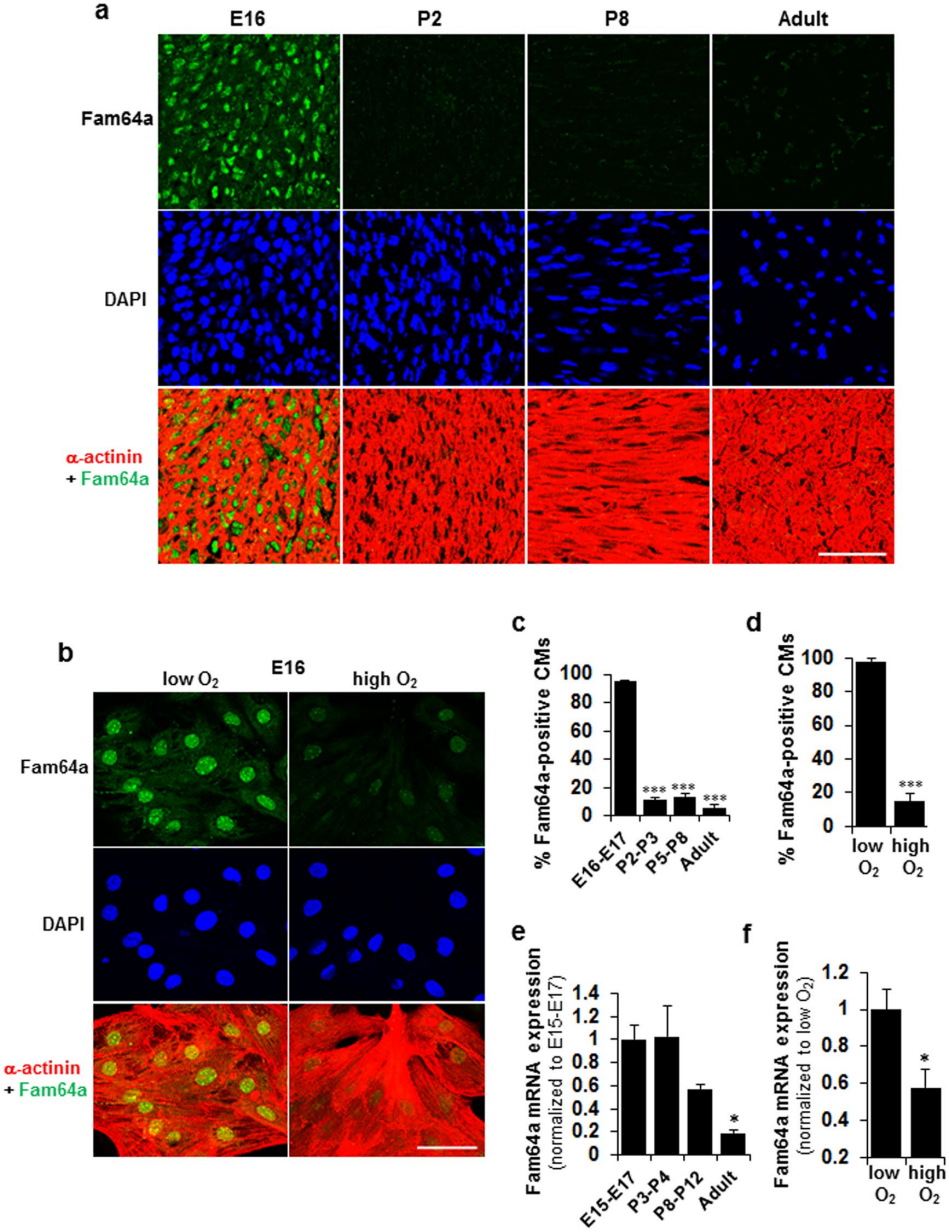
Abundant nuclear Fam64a expression in hypoxic fetal cardiomyocytes (fCMs) is repressed by O_2_ exposure and in postnatal CMs. (**a**,**b**) Immunofluorescence staining of Fam64a and α-actinin in DAPI stained mouse heart sections (**a**) and in E16 fCMs isolated under low O_2_ conditions and cultured under low or high O_2_ conditions (**b**). **(c**,**d**) Percentage of Fam64a-positive fCMs in (**a**) and (**b**), respectively. n = 6–7 microscope images from 3 hearts (**c**) and 10 microscope images from 3 independent experiments (**d**). ^***^ *P* < 0.001 compared to E16–E17 (**c**), or compared to low O_2_ conditions (**d**). (**e**,**f**) qPCR analysis of Fam64a mRNA expression in mouse hearts at indicated stages (**e**) and of E17 fCMs isolated under low O_2_ conditions and cultured under low or high O_2_ conditions (**f**). n = 4 hearts (**e**) and 3 independent experiments (**f**). ^*^ *P* < 0.05 compared to E15–17 (**e**), or compared to low O_2_ conditions (**f**). The reduction in Fam64a mRNA levels in (**e**) was statistically significant with respect to developmental stages, as determined by one-way ANOVA (*P* = 0.008). Error bars = SEM. Scale bars = 50 µm in (**a**,**b**).

We found that Fam64a protein was abundantly expressed in the nuclei of hypoxic fCMs, but this expression was drastically repressed both by O_2_ exposure in culture, and in neonatal CMs after birth following the elevation of O_2_ tension initiated by the onset of breathing (Fig. 4a–d). Expression at the mRNA level was similarly repressed both by O_2_ exposure and after birth (Fig. 4e,f).

### Fam64a expression positively correlates with fCM proliferation

**Figure 5:**
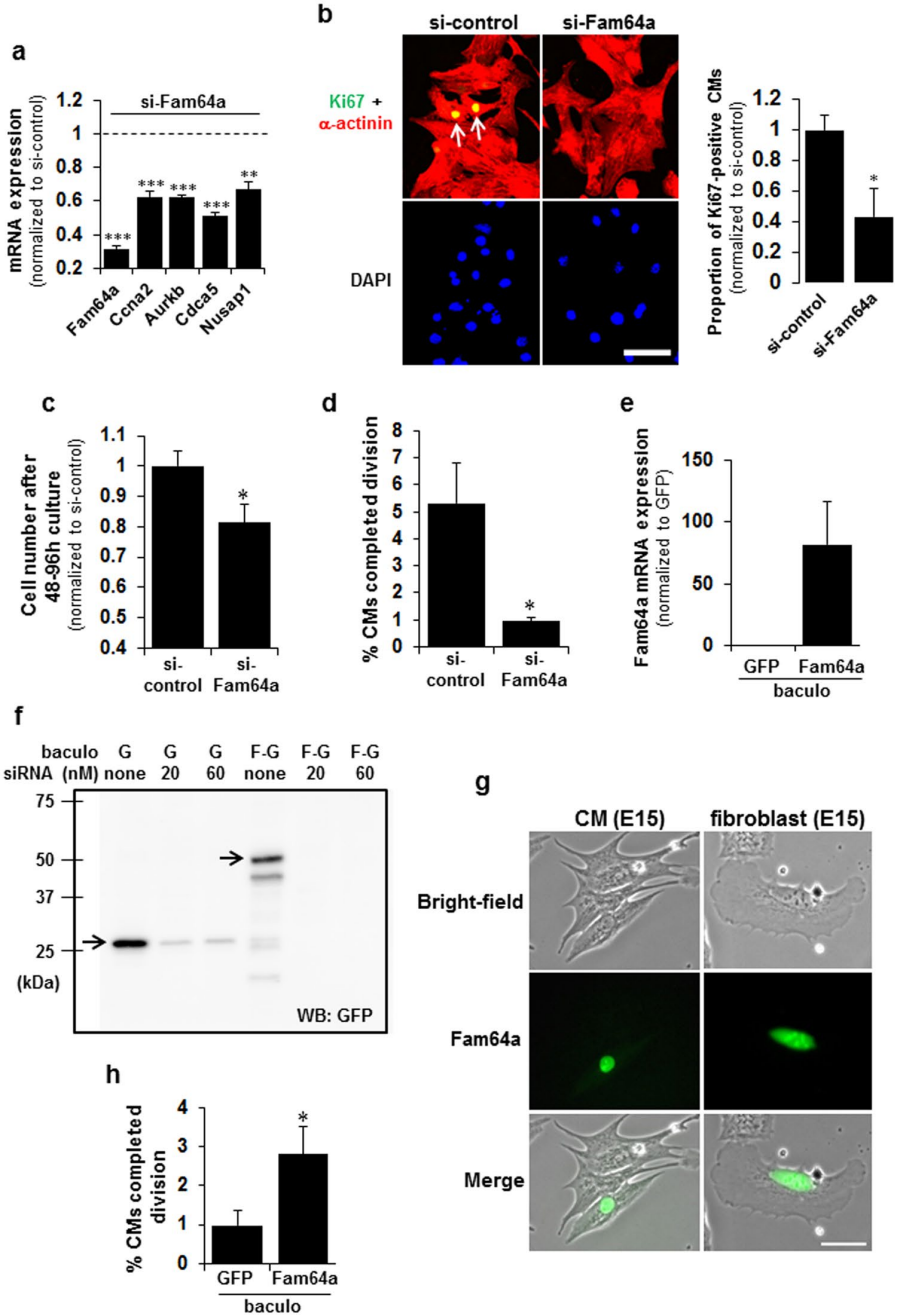
Fam64a expression positively correlates with fetal cardiomyocyte (fCM) proliferation. (**a**) qPCR analysis of mRNA levels of the indicated genes in Fam64a-silenced fCMs (E16–E17) isolated under low O_2_ conditions. n = 3 independent experiments except for Fam64a knockdown evaluated with 2 independent experiments. ^**^ *P* < 0.01 and ^***^ *P* < 0.001 compared to the si-control levels of each gene. (**b**) Immunofluorescence staining for Ki67 observed in α-actinin and DAPI of Fam64a-silenced E16 fCMs isolated under low O_2_ conditions. Arrows denote Ki67-positive CMs. Quantitative analysis of the proportion of Ki67-positive CMs is shown on the right. n = 5 independent experiments. ^*^ *P* < 0.05. (**c**) Proliferative activity of Fam64a-silenced fCMs (E16–E17) evaluated by cell counting. The cells were isolated under low O_2_ conditions and cultured for 48–96 h. n = 5 independent experiments. ^*^ *P* < 0.05. (**d**) Percentage of Fam64a-silenced E16 fCMs that completed cell division, as determined by time-lapse imaging. The cells were isolated under low O_2_ conditions. n = 3 independent experiments and ~700 cells were counted for each condition. ^*^ *P* < 0.05. (**e**) qPCR analysis of Fam64a mRNA expression in fCMs (E15–E16) isolated under high O_2_ conditions and transduced with a Fam64a-GFP-baculovirus or a control GFP-expressing baculovirus. n = 3 independent experiments. (**f**) Immunoblot analysis of fCMs (E15–E16) isolated under high O_2_ conditions and transduced with a Fam64a-GFP-baculovirus or a control GFP-expressing baculovirus (denoted as F-G and G, respectively). A GFP antibody was used as this correctly detected overexpressed Fam64a (28 kDa) as a GFP-tagged protein (28 + 27 kDa). The band specificity was confirmed by co-transfection of siRNA targeting GFP at the indicated doses. (**g**) Localization of transduced Fam64a-GFP protein in fCMs and fibroblasts, as observed in bright-field images. (**h**) Percentage of Fam64a-overexpressed fCMs (E14–E17) that completed cell division, as determined by time-lapse imaging. The cells were isolated under high O_2_ conditions. n = 4–5 independent experiments and ~1800 cells were counted for each condition. ^*^ *P* < 0.05. Error bars = SEM. Scale bars = 50 µm in (**b**) and 30 µm in (**g**).

Fam64a knockdown in fCMs under low O_2_ conditions significantly repressed several major cell cycle-promoting genes (Fig. 5a) and nuclear Ki67 expression (Fig. 5b). In addition, this knockdown significantly inhibited fCM proliferation (Fig. 5c) and markedly inhibited fCM division by 82% (Fig. 5d). The knockdown efficiency was verified by qPCR (Fig. 5a). Next, we established a Fam64a overexpression system in fCMs using the baculovirus-mediated gene transfer via a virus that efficiently infects mammalian cells^16^. The overexpression experiments were performed under high O_2_ conditions to reduce basal Fam64a expression, thereby maximizing the effect of overexpression. The Fam64a mRNA expression increased about 80 fold when compared to control GFP-expressing baculovirus (Fig. 5e). Overexpression of Fam64a (28 kDa) at the protein level was correctly detected as a GFP-tagged protein (28 + 27 kDa) (Fig. 5f). The specificity of the detected band was confirmed by siRNA targeting GFP (Fig. 5f). The tagged Fam64a protein was localized in the nuclei of the fCMs and fibroblasts (Fig. 5g), similarly to endogenous Fam64a (Fig. 4a,b). The Fam64a overexpression significantly enhanced fCM cell division (Fig. 5h). These data show that Fam64a expression level positively correlates with fCM proliferation and suggest a role for Fam64a as a cell cycle promoter in fCMs.

### Fam64a degradation by the APC/C during the metaphase-to-anaphase transition is required for fCM division

**Figure 6:**
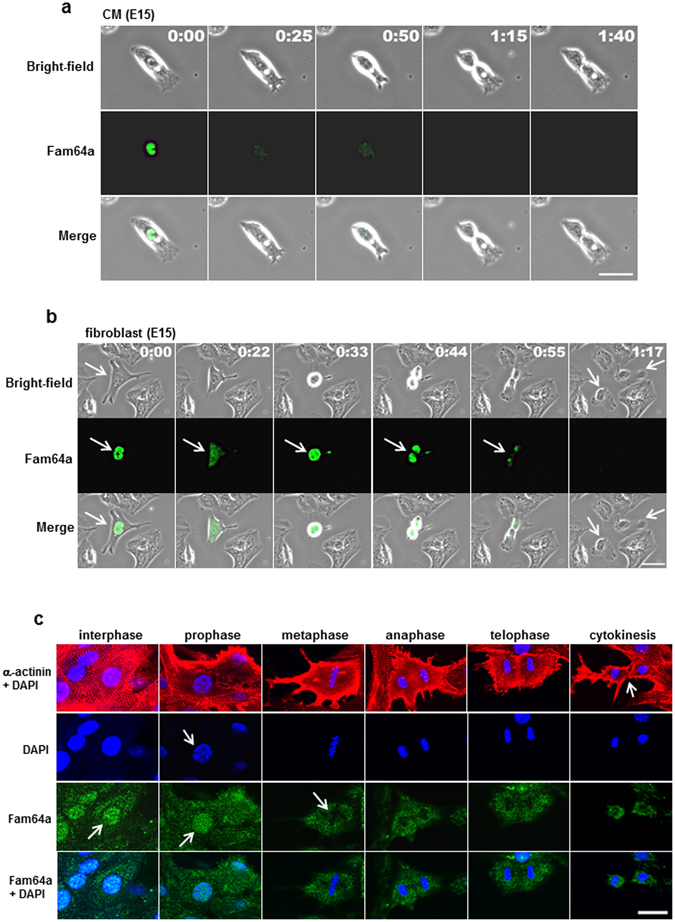
Nuclear Fam64a in dividing fetal cardiomyocytes (fCMs) rapidly disappears during metaphase-to-anaphase transition. (**a**) A representative time-lapse recording of cell division dynamics of E15 fCMs transduced with Fam64a-GFP baculovirus. A steady nuclear signal of Fam64a in an interphase fCM at time 0 rapidly disappeared before the onset of anaphase at 25 min. The fCM subsequently completed mitosis and cytokinesis, but the Fam64a signal never reappeared. (**b**) A representative time-lapse recording of cell division dynamics of E15 fibroblasts transduced with Fam64a-GFP baculovirus. The Fam64a signal disappeared upon completion of mitosis (arrows), which was a later time point than observed for the fCMs in (**a**). (**c**) Isolated E16 fCMs were triple-stained with Fam64a, α-actinin, and DAPI. DAPI staining clearly defined each phase in mitosis, including prophase, metaphase, anaphase, telophase, and subsequent cytokinesis. In interphase CMs, Fam64a expression was restricted to nuclei (arrow) with a faint background signal in the cytoplasm. At this time point, the sarcomere structure was intact, as indicated by α-actinin staining. In prophase, when chromosome condensation was visible as dot-like puncta by DAPI staining (arrow), Fam64a was still expressed in the CM nuclei (arrow). However, during the metaphase-to-anaphase transition, the Fam64a signal rapidly disappeared (arrow) and never reappeared thereafter, while the background signal remained unchanged. Sarcomere structure was grossly perturbed during cell division. Scale bars = 30 µm in (**a**,**b**) and 20 µm in (**c**). In (**a**) and (**b**), the number on each panel indicates the time in (hours: minutes) elapsed from the time indicated in the first panel.

Time-lapse observations showed that the Fam64a-GFP fluorescence in dividing fCMs rapidly disappeared during mitosis (Fig. 6a). In the example shown, the steady nuclear signal of Fam64a observed in an interphase fCM at time 0 rapidly disappeared before the onset of anaphase at 25 min. A movie of the entire time series with additional examples is available in Supplementary Movies 4–6. Notably, all Fam64a-transduced fCMs that underwent cell division showed the same signal disappearance before anaphase, without exception (n = 17 cell division events). By contrast, the disappearance of the Fam64a signal was observed later, at the completion of mitosis, in fibroblasts (Fig. 6b). This disappearance of Fam64a in fCMs was further examined by detailed immunofluorescence analysis in fixed cells triple-stained with Fam64a, α-actinin (as a CM marker), and DAPI (as a marker for each phase in mitosis). This analysis revealed the rapid disappearance of Fam64a during the metaphase-to-anaphase transition, just before the onset of sister chromatid segregation (Fig. 6c). During cell division, the sarcomere structure was grossly perturbed and dynamic changes in cell shape were observed, as reported previously^19^. These observations raised the possibility that Fam64a is degraded by the APC/C, as reported in HeLa cells, although the degradation occurred later, at mitotic exit, in HeLa cells^11^, similarly to the case for fibroblasts in the present study (Fig. 6b). This idea was supported by the fact that relatively few Fam64a-transduced fCMs were found by microscopy (Fig. 5g), despite the 80-fold increase in mRNA level (Fig. 5e), which may reflect a posttranslational degradation.

**Figure 7:**
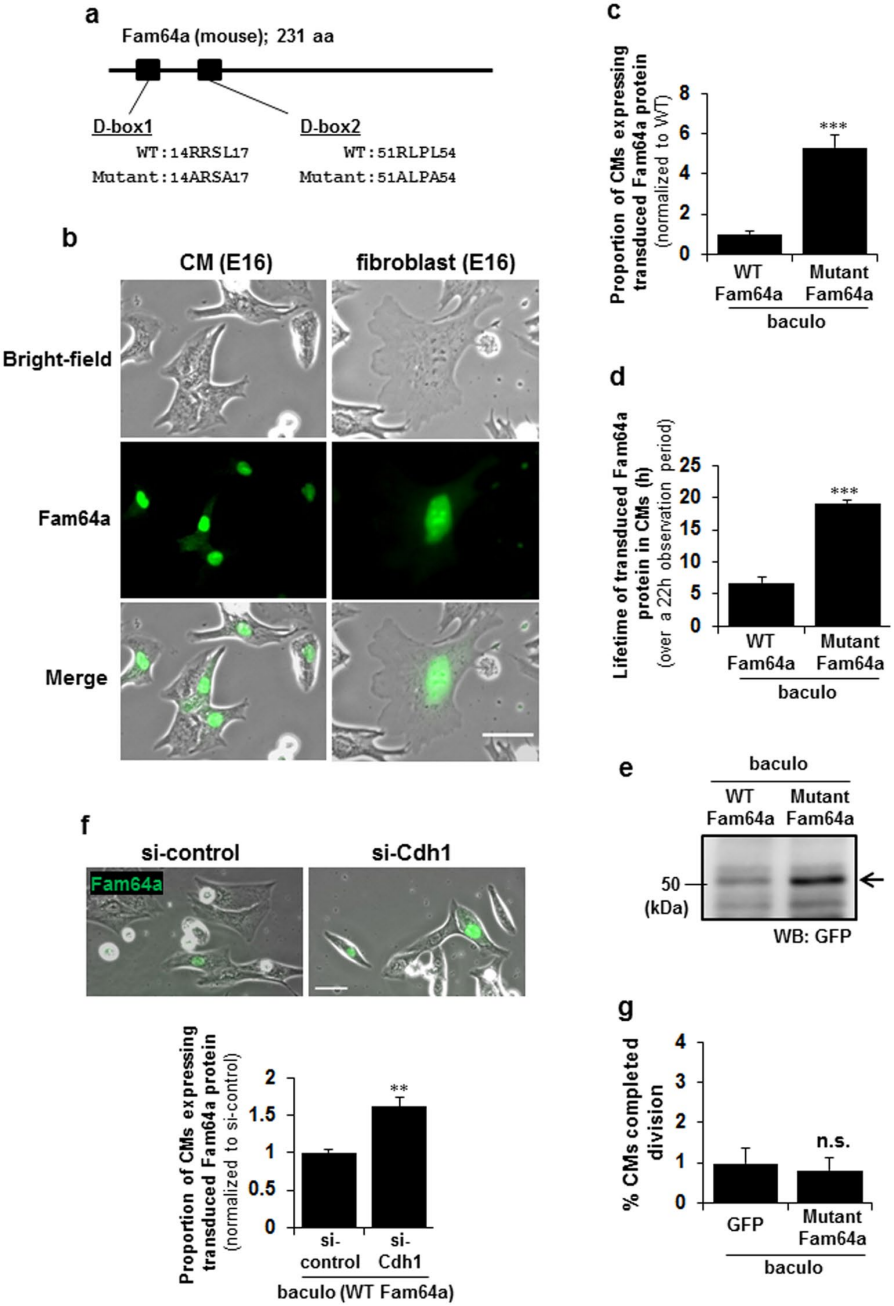
Fam64a degradation by the APC/C during the metaphase-to-anaphase transition is required for fetal cardiomyocyte (fCM) cell division. (**a**) A non-degradable mutant of Fam64a, in which two recognition sites by APC/C complex (D-box1 and D-box2) were mutated (RxxL to AxxA). (**b**) Localization of the transduced mutant Fam64a-GFP protein in fCMs and fibroblasts observed in bright-field images. (**c**) Proportion of fCMs (E15–E17) expressing transduced Fam64a protein out of total fCMs, as detected by GFP fluorescence. n = 3–4 independent experiments, and ~700 cells were counted for each condition. ^***^ *P* < 0.001. (**d**) Lifetime of transduced Fam64a protein in fCMs (E15–E17) over a 22 h observation period, as detected by GFP fluorescence. n = 41 and 66 cells for WT and mutant Fam64a-GFP expressing cells, respectively, from 3–4 independent experiments. ^***^ *P* < 0.001. (**e**) A representative immunoblot analysis of E16 fCMs transduced with WT or mutant Fam64a-GFP baculovirus and then probed with GFP antibody, which correctly detects overexpressed Fam64a (28 kDa) as a GFP-tagged protein (28 + 27 kDa). (**f**) Proportion of Cdh1-silenced E16 fCMs expressing transduced WT Fam64a protein out of total fCMs, as detected by GFP fluorescence. n = 14–40 microscope images from 3 independent experiments, and ~800 cells were counted for each condition. ^**^ *P* < 0.01. (**g**) Percentage of mutant Fam64a-overexpressed fCMs (E15–E17) that completed cell division, as determined by time-lapse imaging. The cells were isolated under high O_2_conditions. n = 5 independent experiments and ~2100 cells were counted for each condition. n.s. = not significant. Error bars = SEM. Scale bars = 30 µm in (**b**,**f**).

The possibility of degradation of Fam64a by the APC/C was examined by transduction of fCMs with a non-degradable mutant of Fam64a, in which two sites recognized by the APC/C complex (D-box1 and D-box2) were mutated (RxxL to AxxA, Fig. 7a)^11^. The mutant Fam64a showed similar nuclear localization in both fCMs and fibroblasts (Fig. 7b), but we detected a greater number of fCMs expressing the transduced Fam64a proteins (Fig. 7b,c) and a longer lifetime of these proteins (Fig. 7d) when compared to wild-type (WT) Fam64a. The WT Fam64a expression persisted for only ~7 h on average during the 22 h observation period, whereas the mutant protein showed almost no signal decay over the 22 h (Fig. 7d). Immunoblot analysis confirmed the Fam64a accumulation (Fig. 7e). Suppression of the activity of the APC/C by knockdown of Cdh1, one of the APC/C activators, led to a similar accumulation of the WT Fam64a (Fig. 7f). These data strongly suggest that the disappearance of the Fam64a signal is the result of APC/C-mediated degradation. Importantly, the mutant Fam64a had no proliferation-promoting effect, unlike the WT Fam64a (Fig. 7g compared to Fig. 5h). Samples overexpressing WT Fam64a also showed some exceedingly bright fCMs that were less prone to divide. These data indicate that an optimum expression of Fam64a and proper degradation of Fam64a by the APC/C during the metaphase-to-anaphase transition are both required for fCM division. Excess accumulation of Fan64a would negatively affect fCM proliferation.

## Discussion

The function of Fam64a in fCMs is currently unknown. We found that Fam64a knockdown inhibits and its overexpression promotes fCM proliferation (Fig. 5), whereas expression of a non-degradable Fam64a mutant does not promote proliferation (Fig. 7). These data indicate that Fam64a serves as a cell cycle promoter, but its mechanism of action is not simple. These findings contrast to those reported for HeLa cells, in which none of the expression modifications, such as knockdown, overexpression, or expression of a non-degradable mutant, appear to change the cell proliferation or the cell cycle gene expressions, and mitosis proceeds normally^11^. The only change observed is a faster progression from metaphase to anaphase following Fam64a knockdown. These results strongly suggest that Fam64a has an indispensable role in CMs, whereas its role is not essential in HeLa cells.

Fam64a degradation occurs at mitotic exit in HeLa cells^11^ and fibroblasts (Fig. 6b), but it occurs earlier, at the metaphase-to-anaphase transition, in fCMs (Fig. 6a,c). The finding that the non-degradable Fam64a mutant cancels the proliferation-promoting effect of WT Fam64a indicates that Fam64a degradation itself is necessary to initiate the sister chromatid segregation at the onset of anaphase in fCMs. By contrast, in HeLa cells, Fam64a was reported to still exist at this stage and it controlled the segregation by interacting with chromatin remodelers to regulate gene expression or chromatin structure^11^. The requirement for Fam64a degradation during the metaphase-to-anaphase transition may be an important checkpoint mechanism specific to CMs, where Fam64a functions to stop sister chromatid segregation until all chromosomes are properly attached to the spindle. In this model, the expression of a non-degradable mutant would prohibit the cell from initiating the segregation, thereby inhibiting proliferation (Fig. 7g). On the other hand, suppressing Fam64a activity by knockdown would impair the proper stop function, resulting in aberrant cell cycle progression, such as premature segregation or mis-segregation, and would eventually lead to cell cycle arrest (Fig. 5a–d). Therefore, fCM proliferation is driven by the following conditions: (1) the expression of an optimum level of Fam64a that is sufficient to trigger the stop function, and (2) degradation of Fam64a at the appropriate time by the APC/C (Fig. 5h). CMs are highly differentiated cells filled with abundant contractile proteins, and must transiently dedifferentiate, with sarcomere disassembly, for cell division (Fig. 6c)^19, 20^, which would cause transient disturbance of the synchronous muscle contraction vital for heart function. Therefore, unlike highly proliferative cells such as HeLa cells and fibroblasts, CMs may need a specific stop mechanism to prevent excessive cell division.

Another important question is which of the two major APC/C activators, Cdc20 or Cdh1, mediates Fam64a degradation in fCMs. In general, Cdc20 is activated at the onset of prometaphase and inactivated during mitotic exit. At that point, Cdh1 is activated and remains active until the onset of the next S phase^14^. In HeLa cells, Fam64a degradation is mediated by both activators *in vitro*, but mainly by Cdh1 *in vivo* at mitotic exit^11^. By contrast, in fCMs, the degradation at the metaphase-to-anaphase transition suggests the involvement of Cdc20. However, Cdh1 would also be implicated because knockdown of Cdh1 affected the accumulation of Fam64a protein (Fig. 7f). This point needs to be addressed in future research.

A recent study showed that proliferation of mouse fCMs during the mid-embryonic stages (E12.5–E14.5) was maintained by Hif-1α, along with other molecules such as Mif^2^. Therefore, we examined the possible involvement of these molecules in Fam64a-mediated fCM proliferation at the late embryonic stage (E16–E18). The high expression of Hif-1α dropped after birth and following O_2_ exposure (Supplementary Fig. 4a), but its knockdown did not affect cell proliferation (Supplementary Fig. 4b) or cell cycle gene expression (Supplementary Fig. 4c). The data for two Hif-1α targets – Mif and prolyl hydroxylase domain protein 3 (Phd3; also known as Egln3) – showed similar responses (Supplementary Fig. 4a–c). These data indicate that Hif-1α, Mif, and Phd3 may play roles, but are not critical, in fCM proliferation at the late embryonic stage. A further examination of the potential involvement of Hif-1α in the transcriptional regulation of Fam64a revealed no evidence that Fam64a transcription is under the control of Hif-1α in fCMs (Supplementary Fig. 4e,f) as determined by both chromatin immunoprecipitation (ChIP) and luciferase reporter assays, even though the Fam64a gene in humans and mice possesses two putative Hif-1α binding sites in its promoter region (5′-GCGTG-3′; Supplementary Fig. 4d). Collectively, the findings of the present study indicated that Fam64a-mediated fCM proliferation at the late embryonic stage is a novel O_2_-dependent system that is independent of Hif-1α. This suggests that fCM proliferation is separately regulated for at least two stages: Hif-1α-driven proliferation at the mid-embryonic stage (E12.5–E14.5) and Fam64a-driven proliferation at the late embryonic stage (E16–E18).

One factor that distinguishes these two stages may be the development of coronary circulation, which is completed at around E16^21, 22^. The formation of coronary circulation greatly facilitates O_2_ delivery to the myocardium, so that fCMs at the mid-embryonic stage would reside in a more severe hypoxic environment than that experienced by the late embryonic fCMs. The decrease in the proliferative activity at the late embryonic stage (E16–E18) compared to the earlier stage (E14–E16), even under the same low O_2_ conditions (Fig. 1c), supports this idea. The mechanisms that regulate this O_2_ alteration and its linkage to the differential regulation of fCM proliferation remain to be addressed.

The Fam64a signal disappearance observed in time-lapse analysis could conceivably be a result of bleaching of GFP signals, but we regard this as unlikely for the following reasons: (1) A similar disappearance was observed in immunofluorescence analysis in fixed cells (Fig. 6c); (2) The non-degradable Fam64a mutant expression (Fig. 7c,d) and Cdh1 knockdown experiments (Fig. 7f) both resulted in the stabilization of the signal; (3) In rare cases, the Fam64a signal, which had once disappeared, reappeared again later. We cannot completely rule out the possibility that the observed disappearance is the result of non-specific dilution of fluorescence signals due to nuclear envelope breakdown at prometaphase; however, the dividing fibroblasts clearly retain those signals after nuclear envelope breakdown (Fig. 6b), which makes this a rather remote possibility.

In summary, we identified a novel O_2_-dependent and Fam64a-mediated system that is essential for fCM proliferation at the late embryonic stage (E16–E18). This system is independent of Hif-1α. Fam64a is abundantly expressed in hypoxic fCM nuclei, but its expression is drastically repressed by O_2_ exposure in cultured cells and after birth in *in vivo* heart tissues. Fam64a is indispensable for fCM proliferation, where its expression and degradation by the APC/C must be balanced for the cell cycle to progress. Postnatal and adult hearts rarely show any expression of Fam64a (Fig. 4a). In addition, the activity of the APC/C declines, as suggested by downregulation of the APC/C activator Cdc20 (Table 3). An important subject for future research will therefore be to test whether the reintroduction of Fam64a and appropriate control of APC/C activities could drive CM proliferation in adult hearts.

## Methods

A detailed Methods section is provided in Supplementary Methods. All animal procedures were approved by the Institutional Animal Care and Use Committee at the Kawasaki Medical School. All experiments were performed in accordance with relevant guidelines and regulations of Kawasaki Medical School.

### Low O_2_ isolation and culture protocol

To mimic intrauterine low O_2_ tension (20–25 mmHg; 2.6–3.2% O_2_) during isolation, all solutions were preconditioned to 2–3% O_2_ by nitrogen gas bubbling. The working space, including CO_2_ incubator and a boxed bench, was also kept strictly in the same low O_2_ conditions by nitrogen or argon gas loading. Under this condition, primary CMs were isolated from ventricles of fetal mice (E12–E18) bred on a C57BL/6 background using a modified protocol for neonatal rat hearts^23^. Briefly, pregnant mice were euthanized with Sevofrane, and fetal heart ventricles were rapidly excised, cut into small pieces, and digested four times with 0.06% trypsin and 0.24 mM EDTA in PBS for 10 min at 37 °C. After 45 min culture to exclude non-CMs, cells were plated onto fibronectin-coated or non-coated culture vessels in DMEM with 5% FBS, and cultured under low (2–3%) or high (21%) O_2_ conditions in a multi-gas incubator (Astec, Japan) at 37 °C with 5% CO_2_. In this study, low and high O_2_ conditions refer to 2–3% and 21% O_2_ tension, respectively. For neonatal mice (postnatal day, P1–P3) or where indicated, isolation was done under high O_2_ conditions (ambient air) without the special procedures described above.

### CM proliferation analysis by FACS

fCMs isolated under low O_2_ conditions were cultured under low or high O_2_ conditions for 96 h. At the start and end of the culture, total cell numbers were counted, and the proportions of CMs and non-CMs were analyzed by FACS to determine the absolute number of each cell type. For FACS analyses, trypsinized cells were fixed with 2% paraformaldehyde, permeabilized, blocked, and stained with primary antibody for sarcomeric α-actinin (Sigma-Aldrich, MO, USA). Mouse IgG_1_ κ isotype control antibody (eBioscience, CA, USA) was used as a negative control. Cells were analyzed with a BD FACSCalibur^™^ (BD Biosciences, Singapore) as described^24^; α-actinin-positive and negative cells were regarded as CMs and non-CMs, respectively.

### Baculovirus-mediated protein expression

Baculovirus that effectively infects mammalian cells was obtained by expressing vesicular stomatitis virus G-protein (VSVG) on the virus envelope^16^. The virus was produced by Bac-to-Bac system (Thermo-Fisher, MA, USA) using the modified donor vector (pFastBac1-VSVG-CMV-WPRE) that was constructed as follows. Cytomegalovirus (CMV) promoter was PCR amplified using pEGFP-N1 (Takara Bio, Japan) as a template, and ligated with pFastBac1. Woodchuck hepatitis virus posttranscriptional regulatory element (WPRE) was PCR amplified from pWPT-GFP (gift from Dr. Didier Trono, Addgene plasmid #12255), and ligated with the above vector. Coding sequence for VSVG and following SV40 polyadenylation signal sequence was amplified from pFBG-CAG^16^, and inserted into downstream of polyhedrin promoter of the above vector to yield the final pFastBac1-VSVG-CMV-WPRE vector. WT and mutant Fam64a sequence was PCR amplified from adult mouse cDNA and synthetic gene fragments (GenScript, NJ, USA), respectively, and inserted into pFastBac1-VSVG-CMV-WPRE. For EGFP-tagged (C-terminus) proteins, EGFP was amplified from pEGFP-N1, and ligated with pFastBac1-VSVG-CMV-WPRE. Subsequently, amplified WT or mutant Fam64a sequence was inserted into EGFP-pFastBac1-VSVG-CMV-WPRE. Baculovirus was produced in Sf9 cells as per the manufacturer’s instructions (Thermo-Fisher). For transduction to fCMs, virus was added to cell suspensions at the time of plating in Minimum Essential Medium (MEM) without serum. After 8–24 h, medium was replaced with MEM with 10% FBS. Primer sequences used for the vector construction were detailed in Supplementary Methods.

### Time-lapse imaging analysis

Isolated fCMs were placed on the stage of the microscopic live cell analyzer (JuLI FL; NanoEnTek, Korea), which was accommodated in a multi-gas incubator. Time-lapse imaging of fCM division under low or high O_2_ conditions was recorded at 10 min intervals by phase contrast microscopy, which was initiated at 9–11 h after plating, and continued for ~20 h. Complete fCM division events, in which mitosis was followed by cytokinesis, resulting in the generation of two daughter cells (examples shown in Fig. 2a–b), were manually counted and defined as the percentage of total fCMs in the imaging field. For baculovirus-transduced fCMs, time-lapse experiments were performed with inverted fluorescence microscope (BZ-X710, Keyence, Japan) or confocal microscope (FV1000, Olympus, Japan) equipped with a stage incubation system.

### DNA microarray

For the comparison between neonates and fetuses (array #1, Fig. 3a), CMs were isolated from E16 fetal hearts under low O_2_ conditions, or from P2–P3 neonatal hearts under high O_2_ conditions, and total RNA was immediately obtained from CM pellets (with no further culturing) using RNeasy Plus Mini Kit (QIAGEN, CA, USA). This prevented contamination from non-CMs in cases where the RNA was obtained from whole heart. For the analysis of fCM exposure to O_2_ (array #2, Fig. 3a), E18 fCMs isolated under low O_2_ conditions were separately cultured under low/high O_2_ conditions for 48 h and then total RNA was obtained. For DNA microarray analyses, the 3D-Gene Mouse Oligo chip 24k (Toray Industries, Japan) was used (23,522 distinct genes). Total RNA was labelled with Cy5 (one color design; array #1) or Cy3/Cy5 (two color design; array #2) using the Amino Allyl MessageAMP II aRNA Amplification Kit (Applied Biosystems, CA, USA), and hybridized for 16 h. Hybridization signals were scanned by 3D-Gene Scanner and processed by 3D-Gene Extraction software (Toray Industries). The raw data from each spot were normalized by subtraction, with the mean intensity of the background signal determined by all blank spot signal intensities with 95% confidence interval. The raw data intensities greater than two standard deviations of the background signal intensities were considered to be valid. Detected signals for each gene were normalized by global normalization methods. Genes with normalized ratios of > 2.0 and < 0.5 were defined, respectively, as upregulated and downregulated genes. Pathway analyses were performed with GenMAPP software version 2.1 (MAPP Finder at http://www.genmapp.org/, Gladstone Institutes, University of California at San Francisco) with threshold ratios of 2.0 (up)/0.5 (down) in array #1 and 1.5 (up)/0.67 (down) in array #2.

### Gene silencing by siRNA

Small interfering RNA (siRNA)-mediated knockdown of the specific genes was performed in isolated fCMs using Lipofectamine^®^ RNAiMAX or Lipofectamine^®^ 2000 (Thermo-Fisher) as per the manufacturer’s protocol. Cell density, the timing of knockdown, the amount of siRNA (mostly 15–30 nmol/L), and the timing of evaluation were optimized in each experiment. siRNAs used were detailed in Supplementary Methods.

### Immunofluorescence

For tissue analyses, frozen heart sections embedded in OCT compound (Tissue-Tek^®^; Sakura, UAE) were cut at 8 µm sections with a cryostat (Leica, Germany), permeabilized, blocked with Blocking-One (Nacalai Tesque, Japan), and double-labeled with primary antibodies for sarcomeric α-actinin (Sigma-Aldrich) and Fam64a. The latter antibody was raised against a synthetic peptide corresponding to residues 102–114 of mouse Fam64a (CQSGTKWLMETQV). Samples were then labeled with fluorochrome-conjugated secondary antibodies (Thermo-Fisher) as described^25, 26^. When necessary, DAPI staining for DNA or phalloidin staining for F-actin filaments was also performed. The same protocol was applied for cultured cells, except that fixation with 4% paraformaldehyde was first performed before permeabilization. Primary antibodies used were against Ki67 (Abcam), phospho-histone H3 at Ser-10 (EMD Millipore, MA, USA), sarcomeric α-actinin (Sigma-Aldrich), and Fam64a (Bioss Antibody, GA, USA). Another Fam64a antibody, which was raised as stated above, was also used. When using mouse-derived antibodies, the Mouse on Mouse (M.O.M.^™^) Basic Kit (Vector, CA, USA) was used. Cells or sections covered with fluorescence mounting medium (DAKO, CA, USA) were examined using a confocal system mounted on a IX81 inverted microscope (Olympus).

### Immunoblotting

Hearts were collected from mice and snap frozen in liquid nitrogen, minced, and homogenized using a Kinematica^™^ Polytron^™^ homogenizer (PT1600E; Fisher Scientific, MA, USA) in lysis buffer (10 mM Tris-HCl, pH 7.5, 150 mM NaCl, 0.5 mM EDTA, 10 mM NaF, and 0.5% Triton X-100), RIPA buffer (Thermo-Fisher), or M-PER buffer (Thermo-Fisher) in the presence of protease and phosphatase inhibitor cocktail (Thermo-Fisher or Roche, CH). For cultured CMs, harvested cell pellets were lysed in the same buffer and processed as was done for heart tissues. Lysates were centrifuged at 14,000 × *g* and supernatants were used as the whole protein extract. In some samples, nuclear extracts were subsequently obtained with nuclear extraction buffer (20 mM Tris-HCl, pH 7.5, 20% glycerol, 0.5 M NaCl, 1.5 mM MgCl_2_, 0.1% Triton X-100, and 1 mM DTT) for Hif-1α detection. After quantifying protein yield, equal amount of proteins were separated by SDS-PAGE (Mini-PROTEAN^®^ TGX; Bio-Rad, CA, USA), transferred onto PVDF membranes (GE Healthcare), blocked with BLOCK-ACE (DS Pharma, Japan) with 0.5% BSA or 5% nonfat milk, and probed with primary antibodies, followed by secondary horseradish peroxidase (HRP)-conjugated IgG (GE Healthcare), and finally visualized by enhanced chemiluminescence (Western Lightning ECL-Pro; PerkinElmer, OH, USA) using a LAS4000mini luminescent image analyzer (GE Healthcare). Densitometry analysis was performed using Image J. Primary antibodies used were detailed in Supplementary Methods.

### Quantitative PCR (qPCR)

Hearts were collected from mice, cut into small pieces, and immediately immersed in RNAlater^®^ Stabilization Reagent (QIAGEN). The stabilized tissues were homogenized with a Micro Smash^™^ homogenizer (MS-100R; Tomy, Japan), and total RNA was isolated using RNeasy^®^ Plus Mini Kit (QIAGEN), RNeasy^®^ Fibrous Tissue Mini Kit (QIAGEN), or ISOGEN system (Nippon Gene, Japan). For cultured CMs, harvested cell pellets in appropriate buffer were processed as was done for heart tissues. After assessing RNA yield and quality using NanoDrop spectrophotometer (ND-1000; Thermo-Fisher), RNA samples were reverse-transcribed with PrimeScrip RT Master Mix (TaKaRa Bio), and quantitative real-time PCR was performed using TaqMan^®^ Fast Advanced Master Mix in a StepOnePlus^™^ real-time PCR system (Applied Biosystems). TaqMan^®^ gene expression assays used were detailed in Supplementary Methods. Quantification of each mRNA was carried out with *Actb* (#Mm00607939_s1), *Rn18S* (#Mm03928990_g1), *18 S* (#Hs99999901_s1), or *Ubc* (#Mm01198158_m1) as reference genes using the ΔΔCT method.

### Statistics

All data were expressed as mean plus or minus standard error of the mean (SEM). For comparisons between two groups, Student’s two-tailed paired *t*-test was used to determine statistical significance. For comparisons among multiple groups, one-way analysis of variance (ANOVA) was used with Bonferroni’s post hoc test. *P* < 0.05 was considered statistically significant. Significance levels were indicated as ^*^ *P* < 0.05, ^**^ *P* < 0.01, and ^***^ *P* < 0.001.

### Data availability

All data generated or analysed during this study are included in this published article (and its Supplementary Information files).

## Electronic supplementary material

Supplementary Info

Movie 1

Movie 2

Movie 3-1

Movie 3-2

Movie 4-1

Movie 4-2

Movie 5

Movie 6-1

Movie 6-2

Supplementary Dataset 1

Supplementary Dataset 2
