# Supplementary material for: Fam64a is a novel cell cycle promoter of hypoxic fetal cardiomyocytes in mice
Source: Sci Rep. 2017 Jun 30;7:4486. doi: 10.1038/s41598-017-04823-1 (PMC5493652; doi:10.1038/s41598-017-04823-1)
Supplement: Supplementary file 1 — Supplementary Info [file 41598_2017_4823_MOESM1_ESM.pdf]

Fam64a is a novel cell cycle promoter of hypoxic fetal cardiomyocytes in mice.

*Ken Hashimoto, Aya Kodama, Takeshi Honda, Akira Hanashima, Yoshihiro Ujihara,
Takashi Murayama, Shin-ichiro Nishimatsu, Satoshi Mohri

Supplementary Figures 1-4

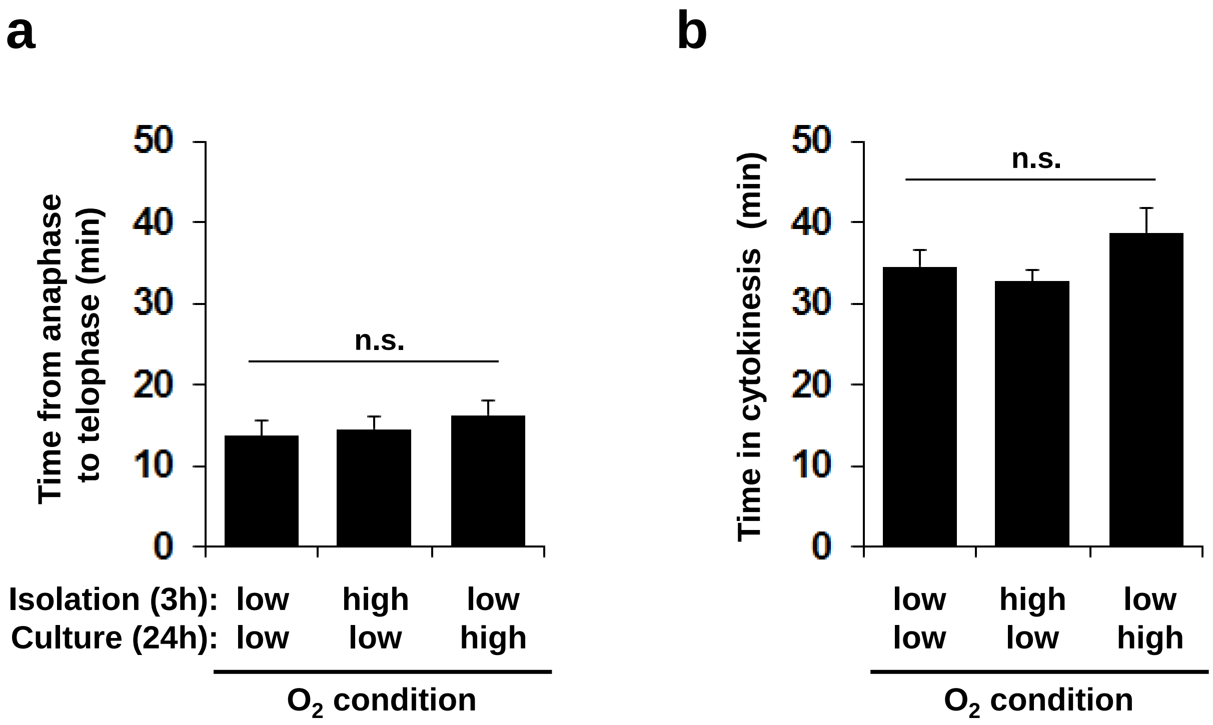

Supplementary Fig. 1 Kinetic analysis of fCM division in time-lapse imaging

(a, b) Time from anaphase to telophase **(a)** and in cytokinesis **(b)** in dividing fCMs (E16–E17) under the indicated isolation and culture conditions. n=8-11 dividing fCMs. n.s.=not significant. Error bars=SEM.

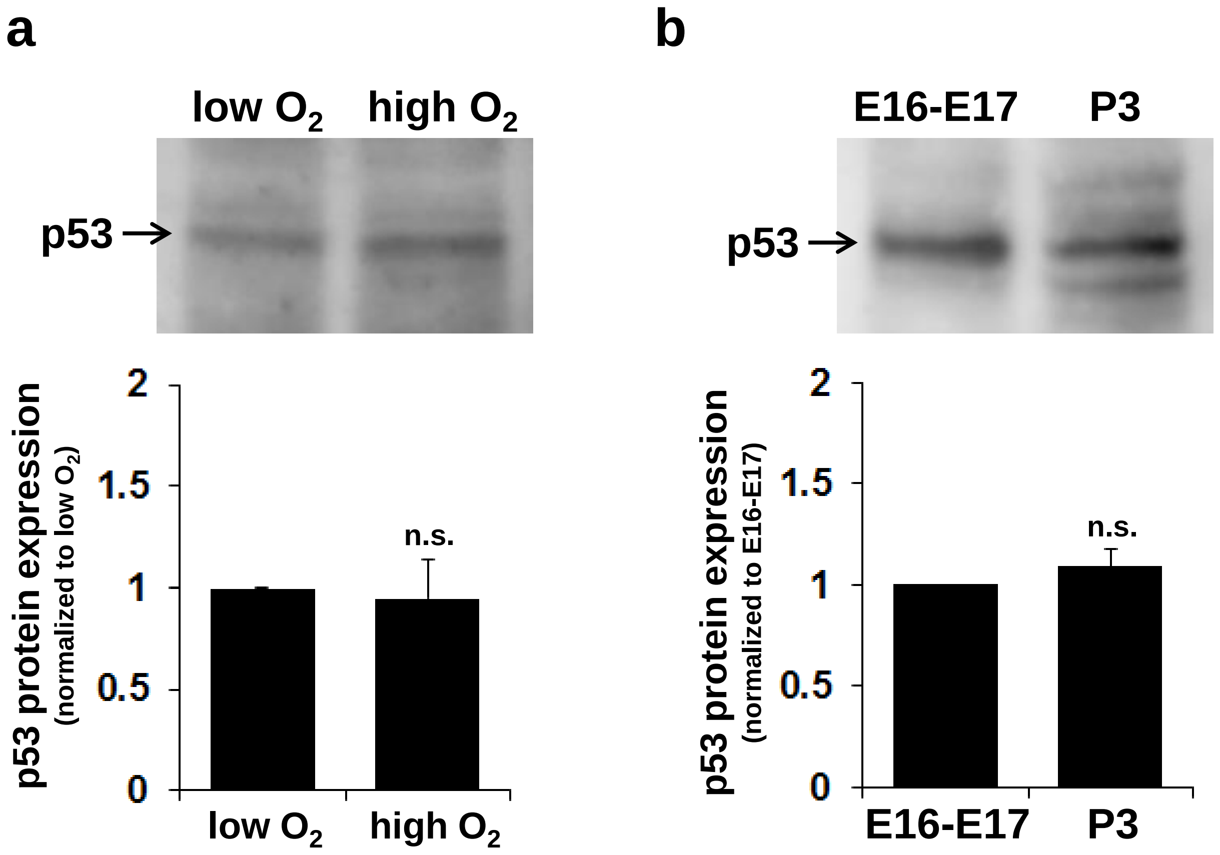

a
low O2
high O2
p53
2
1.5
1
0.5
0
p53 protein expression
(normalized to low O2)
n.s.
low O2
high O2
b
E16-E17
P3
p53
2
1.5
1
0.5
0
p53 protein expression
(normalized to E16-E17)
n.s.
E16-E17
P3

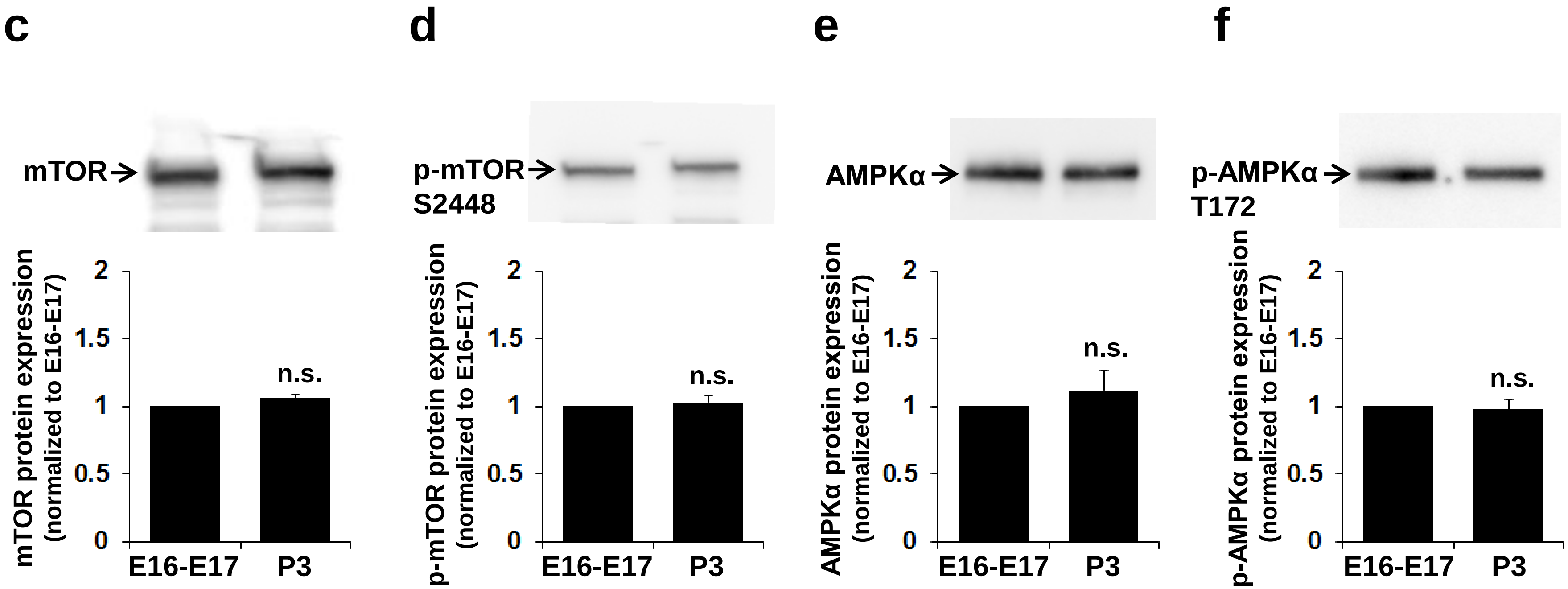

Supplementary Fig. 2 p53, mTOR, and AMPKα are not involved in low O_2 condition-induced fCM proliferation
(a, b) Immunoblotting for p53 in low O_2-isolated fCMs (E16–E17) cultured under low or high O_2 conditions **(a)**, or in mouse hearts around birth **(b)**. **(c–f)** Immunoblotting for mTOR **(c)**, phosphorylated m-TOR at Ser-2448 **(d)**, AMPKα **(e)**, and phosphorylated AMPKα at Thr-172 **(f)** in mouse hearts around birth. n=2 independent experiments **(a)** and 4–6 hearts **(b-f).** n.s.=not significant. Error bars=SEM.

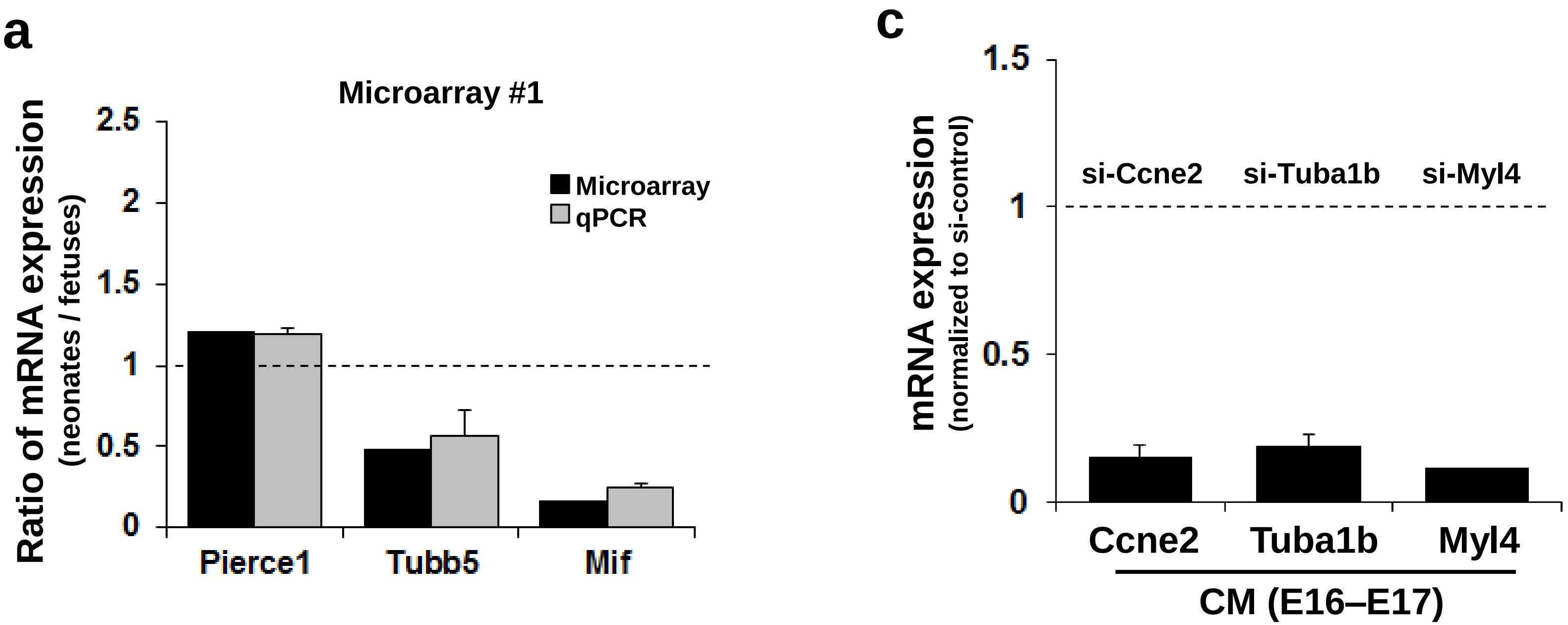

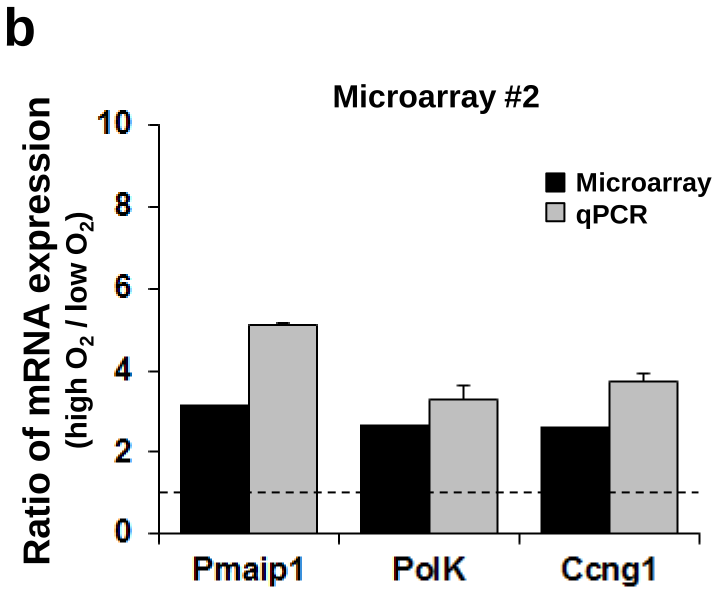

Supplementary Fig. 3 Validation of microarray data and siRNA-mediated knockdown efficiency

(a) For randomly selected three genes (Pierce1, Tubb5, and Mif), data of microarray #1 was verified by qPCR. Data are expressed as an expression ratio of neonates (P2–P4) compared to fetuses (E15–E17). **(b)** For randomly selected three genes (Pmaip1, PolK, and Ccng1), data of microarray #2 was verified by qPCR. Data are expressed as an expression ratio of high O_2- compared to low O_2-cultured fCMs (E16–E18), in which isolation was done under low O_2 conditions. qPCR data in **(a, b)** are expressed as mean plus SEM from 3 independent experiments. **(c)** Knockdown efficiency of siRNA-mediated gene silencing was verified by qPCR in low O_2-isolated and low O_2-cultured fCMs (E16–E17). Representative data for several genes (Ccne2, Tuba1b, and Myl4) are shown.

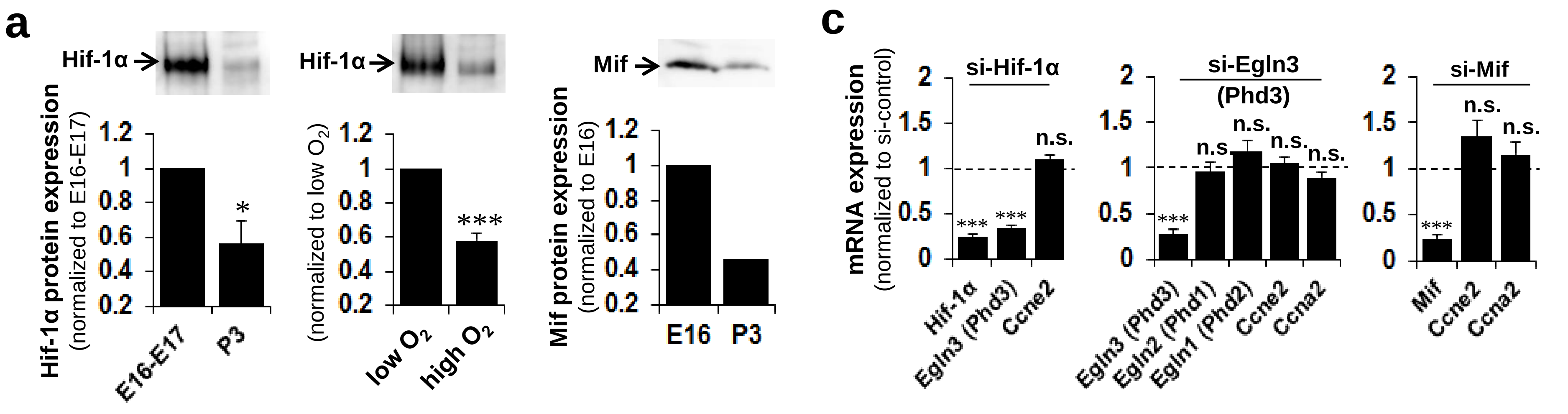

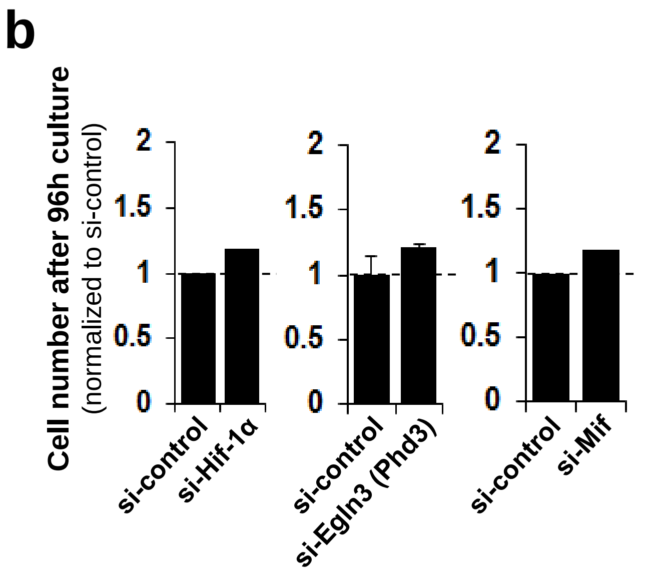

Supplementary Fig. 4 Fam64a-mediated fCM proliferation at late-embryonic stage is independent of Hif-1α

(a) Immunoblotting for Hif-1α (left) and Mif (right) in mouse hearts around birth, or for Hif-1α (middle) in low O_2-isolated fCMs (E16–E18) cultured under low or high O_2 conditions. n=4 hearts (left) and 3 independent experiments (middle). In some samples, nuclear fractions were used to clearly detect Hif-1α. $^{*}P<0.05$ and $^{***}P<0.001$. **(b)** In low O_2-isolated fCMs, Hif-1α (left), Phd3 (middle), or Mif (right) was knocked down, cells were cultured for 96h, and proliferative activity was evaluated by cell counting. **(c)** qPCR analyses for mRNA levels of the indicated genes, in Hif-1α- (left), Phd3- (middle), or Mif- (right) silenced fCMs (E16–E17), in which isolation was done in low O_2 conditions. n=3–4 independent experiments. $^{***}P<0.001$ compared to si-control levels of each gene. **(d)** Nucleotide sequence of human *FAM64A* gene promoter region. Two candidate Hif-1α consensus binding sites 5'-GCGTG-3' were shown in red. Six primers for ChIP assays (P1–P6, underlined) were designed to encompass a range of human *FAM64A* promoter. TSS; transcription start site. **(e)** ChIP assays for Hif-1α or Hif-2α were performed in HEK293T cells and fetal mouse heart tissues (E16–E17). Fold enrichment over control IgG was calculated for designed primers (human; P1–P6, mouse; M1–M5). n=5–14 independent experiments. **(f)** Luciferase reporter assays were performed using human *FAM64A* promoter sequence in HEK293T cells (left) and fCMs (middle and right). Hif-1α protein was stabilized through PHD inhibitor DMOG under high O_2 conditions (left and middle), or was silenced by siRNA-mediated knockdown under low O_2 conditions (right). n=2–4 independent experiments. n.s.=not significant. Error bars=SEM.

d

```
TCCTTTCCTTGGAAATCACTGAGGCCAAAGGAGTGGGTGAGACCACCCAGAAACGGAGAGGGCTGAAGACAGAGTCTCAAGAAGCCATCATCTCAGGCTCTTGTGCGGAGAAGTAACAAAGAAAC
→ P1                                                                                                                   → P2

CAGCCGGGGGAGTGGGCAGCACTGCTCTGAGGTCACCGAAAGGAGAAAGATGAGCTGGAGAGAGCTTTATAATGGCTGCATTTGTTTGGATGCTAAGGAGAAGAGAGGCAGGCATTCCGCTGGAG
                                  P1←                                                        → P3

AGAGGGAGGGATGCGTGGGGCAAGGCGGGGGCGGGGAGCCGGTGTAGACGGGTGGAGGGCAGACCGGTACTCTGGTCTATCCCCGCTTCCGGGACGCGTTTTCTAGGGAGGGCGGAGCGTGGATG
                                                                          P2←                   → P4

GCGAGTATGGGGAACTCAGAGAGGGACAGTGTCTTGTCACAGGAAGGGAGGGTCAGGAGGGAAATTTGAAAGAAAACGTCTCTGGGAACCCAGGGCTCTGCACTGCCAGTCCCTGGCTCCTTCTG
                           P3←                                          → P5

ACAAAACCACTGAGCAAGAAGGAAAAGCCAGACCCCTTGGCAGGATGAGAGGGGTCTCTGCCCGCTGCCGGGCCTGGGGCCTGGGGCCTGAGCCAGGAGGCCTAGGTCCCAGGACCTCCGCCTCC
                         P4←

ACCCCATGGGTCAGAGCAGCCTGAACCCTGTGCGCGCTCCAGGGAGGGGACCCGGTGTGCGATGCGCGGGGGCTGGGCTAGCAAGGGCAGTGGCGCGAGCGGGACGCAGGATACAAAGCCCGGAG
     → P6                   P5←

CCACGAGGCTGGCGGCGGGGCGGGGGCCGGAGCGATGAATGGGCACGGCCGGGCCAATCCAAGCGCAGCATTTGCAGCCGCGAGAGAGGGAGCCAATCGGATCCCCGGGGCGGTGGCGGTGGCGG
                                                                    P6←

GGGCGGGGACGGAACCAATCAGCTGTGAGGACGGGGCCGGGGGCGGAGCCG|CGCAGA
                                                   |→ TSS
```

e

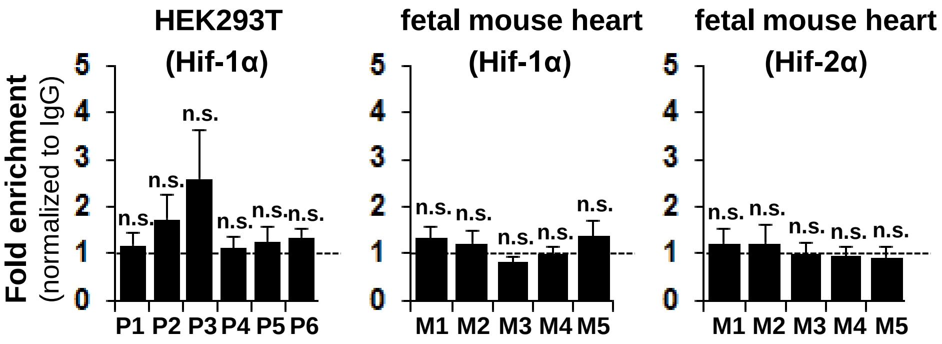

f

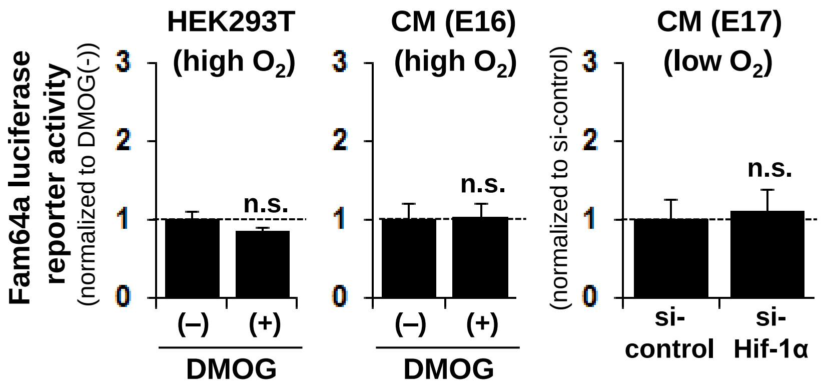

Fam64a is a novel cell cycle promoter of hypoxic fetal cardiomyocytes in mice.

*Ken Hashimoto, Aya Kodama, Takeshi Honda, Akira Hanashima, Yoshihiro Ujihara,

Takashi Murayama, Shin-ichiro Nishimatsu, Satoshi Mohri

Supplementary Methods

All animal procedures were approved by the Institutional Animal Care and Use Committee at the Kawasaki Medical School. All experiments were performed in accordance with relevant guidelines and regulations of Kawasaki Medical School.

Low O_2 isolation and culture protocol.

To mimic intrauterine low O_2 tension (20–25 mmHg; 2.6–3.2% O_2) during isolation, all solutions were preconditioned to 2–3% O_2 by nitrogen gas bubbling and were then kept until further use. The working space, including the CO_2 incubator and a boxed bench (AS-600P; As-One, Japan) was also kept strictly in the same low O_2 conditions by nitrogen or argon gas loading. Under this condition, primary CMs were isolated from ventricles of fetal mice (embryonic day, E12–E18) bred on a C57BL/6 background by gentle trypsin treatment, using a modification of the protocol used for neonatal rat hearts[1]. Pregnant mice were euthanized with an overdose of Sevofrane. Delivered fetuses were decapitated, and ventricles were rapidly excised, cut into small pieces, and washed once with PBS followed by gentle shaking. The tissues were then digested four times with 0.06% trypsin and 0.24 mmol/L EDTA in PBS for 10 min at 37°C, again with gentle agitation. Cell suspensions were then resuspended in DMEM with 5% FBS and cultured for 45 min to exclude non-CMs (mostly fibroblasts), which preferentially adhered to the plates. Supernatants containing CMs were filtered through a 100 µm cell strainer (Falcon™, BD Biosciences, NJ, USA), and the cell pellets were resuspended in fresh DMEM with 5% FBS, and plated onto fibronectin-coated or non-coated culture vessels. This protocol took ~3h and consistently yielded >95% pure CMs with a few contaminating fibroblasts, which were evaluated by FACS as positive sarcomeric α-actinin expression in CMs (Fig. 1a). Isolated CMs were subsequently cultured under low (2–3%) or high (21%) O_2 conditions in a

multi-gas incubator (APM-30DR, Astec, Japan) at 37 °C with 5% CO_2. In this study, low or high O_2 conditions refer to 2–3% or 21% O_2 tension, respectively. For neonatal mice (postnatal day, P1–P3) and in some experiments using fetuses or where indicated, isolation was conducted under high O_2 conditions (ambient air) without the special procedures described above.

CM proliferation analysis by FACS.

fCMs isolated under low O_2 conditions were subsequently cultured under low or high O_2 conditions for 96h. At the start and end of the culture, total cell numbers were manually counted, and the proportions of CMs and non-CMs in the same sample were analyzed by FACS. This allowed us to determine the absolute number of each cell type (CMs and non-CMs) at the start and end of the culture. For FACS analyses, trypsinized cells were fixed with 2% paraformaldehyde, permeabilized with 0.5% Tween-20, blocked with 5% BSA, and labelled with primary antibody for sarcomeric α-actinin (clone EA-53, Sigma-Aldrich, MO, USA) followed by secondary Alexa 488-conjugated goat anti-mouse IgG (Thermo-Fisher, MA, USA). Negative control samples were labelled with Alexa 488-conjugated mouse IgG_1 κ isotype control antibody (eBioscience, CA, USA). Cells were analyzed with a BD FACSCalibur™ (BD Biosciences, Singapore) as described[2]; α-actinin-positive cells were regarded as CMs and α-actinin-negative cells as non-CMs.

Baculovirus-mediated protein expression in CMs.

Baculovirus that effectively infects mammalian cells was obtained by expressing vesicular stomatitis virus G-protein (VSVG) on the virus envelope[3]. The virus was produced by Bac-to-Bac system (Invitrogen) using the modified donor vector (pFastBac1-VSVG-CMV-WPRE) that was constructed as follows. Cytomegalovirus (CMV) promoter was amplified by PCR with primers (5'-AAGGATCCTAGTTATTAATAGTAATCAAT-3' and 5'-CCGAATTCTTGGAAGCTTTAGCGCTAGCGGATCTGACGG-3') using pEGFP-N1 (Clontech) as a template, digested by BamHI/EcoRI and ligated with pFastBac1 (Invitrogen) in which existing HindIII and EcoRV sites have been deleted. Woodchuck hepatitis virus posttranscriptional regulatory

element (WPRE) was PCR amplified from pWPT-GFP (gift from Dr. Didier Trono, Addgene plasmid # 12255) with primers (5'-GGCTCGAGAATTGATATCTCAACCTCTGGATTACAAAAT-3' and 5'-TTGGTACCAATTCCCGATGCGGGGAGGCG-3'), digested by XhoI/KpnI and ligated with the above vector. Coding sequence for VSVG and following SV40 polyadenylation signal sequence was amplified with primers (5'-CCGGATCCATGAAGTGCCTTTTGTACTTA-3' and 5'-CCGAATTCAATTCGTACGCGGATCTCCTAGGCTCAAGCA-3') from pFBG-CAG[3], digested by BamHI/BsiWI and inserted into downstream of polyhedrin promoter of the above vector to yield the final pFastBac1-VSVG-CMV-WPRE vector. WT or mutant Fam64a sequence was PCR amplified from adult mouse cDNA or artificial synthetic gene fragment (GenScript, NJ, USA) with primers (5’-TATACGCGTATGGCGTCTCAGTGGCAG-3’ and 5’-TATGCGGCCGCTCATTCACGGATAAGGGA-3’), digested by MluI/NotI and inserted into pFastBac1-VSVG-CMV-WPRE vector. For EGFP-tagged (C-terminus) proteins, EGFP was amplified by PCR with primers (5'-ATACTCGAGATGGTGAGCAAGGGCGAG-3' and 5'-ATAGATATCTTACTTGTACAGCTCGTC-3') using pEGFP-N1 (Clontech) as a template, digested by XhoI/EcoRV and ligated with pFastBac1-VSVG-CMV-WPRE vector. Subsequently, WT or mutant Fam64a sequence was PCR amplified from adult mouse cDNA or artificial synthetic gene fragment (GenScript) with primers (5’-TATACGCGTATGGCGTCTCAGTGGCAG-3’ and 5’-ATACTCGAGTTCACGGATAAGGGAGAC-3’), digested by MluI/XhoI and inserted into EGFP-pFastBac1-VSVG-CMV-WPRE vector. Baculovirus was produced in Sf9 cells according to the manufacturer's instructions (Invitrogen), and stored at 4 °C until use. For transduction to fCMs, virus was added to cell suspensions at the time of plating in Minimum Essential Medium (MEM) without serum. After 8–24 h, medium was replaced with MEM with 10% FBS.

Time-lapse imaging analysis of CM cell division.

Isolated fCMs were placed on the stage of the microscopic live cell analyzer (JuLI FL; NanoEnTek, Korea), which was accommodated in a multi-gas incubator. Time-lapse imaging of fCM division under low or high O_2 conditions was recorded at 10 min intervals by phase contrast microscopy,

which was initiated at 9–11h after plating, and continued for ~20 h. This time frame was optimal to image fCM division. Complete fCM division events, in which mitosis was followed by cytokinesis, resulting in the generation of two daughter cells (examples shown in Fig. 2a–b), were manually counted and defined as the percentage of total fCMs in the imaging field. A few contaminating non-CMs (mostly fibroblasts) were easily excluded by their morphology, smaller cell size, and active migration. In addition, post-imaging samples were fixed with 4% paraformaldehyde and immunostained for sarcomeric α-actinin to confirm that the dividing cells were unequivocally CMs (Fig. 2b, c). For baculovirus-transduced fCMs, time-lapse experiments were performed with inverted fluorescence microscope (BZ-X710, Keyence, Japan) equipped with a stage incubation system (INUG2-KIW, Tokai Hit, Japan) or confocal microscope (Fluoview™ FV1000; Olympus, Japan) equipped with a stage incubation system (Chamlide TC, Live Cell Instrument, Korea).

DNA microarray.

For the comparison between neonates and fetuses (array #1, Fig 3a), CMs were isolated from E16 fetal hearts under low O_2 conditions, or from P2–P3 neonatal hearts under high O_2 conditions, and total RNA was immediately obtained from the CM pellets (with no further culturing) using the RNeasy Plus Mini Kit (QIAGEN, CA, USA). This prevented contamination from non-CMs in cases where the RNA was obtained from the whole heart. For the analysis of fCM exposure to O_2 (array #2, Fig 3a), E18 fCMs isolated under low O_2 conditions were separately cultured under low or high O_2 conditions for 48h and then total RNA was obtained, also using the RNeasy Plus Mini Kit (QIAGEN). For the oligo DNA microarray analyses, the 3D-Gene Mouse Oligo chip 24k (Toray Industries, Japan) was used (23,522 distinct genes). For efficient hybridization, this microarray is 3-dimensional, with a well as the space between the probes and cylinder-stems, and with 70mer oligonucleotide probes on the top. Total RNA was labelled with Cy5 (one color design; array #1) or Cy3/Cy5 (two color design; array #2) using the Amino Allyl MessageAMP II aRNA Amplification Kit (Applied Biosystems, CA, USA), and hybridized for 16h. The hybridization was performed as per the manufacturer's protocol (www.3d-gene.com). Hybridization signals were scanned using 3D-Gene Scanner (Toray Industries)

and processed by 3D-Gene Extraction software (Toray Industries). The raw data from each spot were normalized by subtraction, with the mean intensity of the background signal determined by all blank spot signal intensities with 95% confidence interval. The raw data intensities greater than two standard deviations of the background signal intensities were considered to be valid. Detected signals for each gene were normalized by global normalization methods. Genes with normalized ratios greater than 2.0 or less than 0.5 were defined, respectively, as commonly upregulated or downregulated genes. Pathway analyses were performed with GenMAPP software version 2.1 (MAPP Finder at http://www.genmapp.org/, Gladstone Institutes, University of California at San Francisco) with threshold ratios of 2.0 (up)/0.5 (down) in array #1 and 1.5 (up)/0.67 (down) in array #2.

Gene silencing by siRNA.

Small interfering RNA (siRNA)-mediated knockdown of the specific genes was performed in isolated fCMs using Lipofectamine® RNAiMAX or Lipofectamine® 2000 (Thermo-Fisher) as per the manufacturer's protocol. Cell density, the timing of knockdown, the amount of siRNA (mostly 15–30 nmol/L), and the timing of evaluations were optimized in each experiment. The siRNAs for *Birc5*, *Ankrd1*, *Acta2*, *Rpl41*, *Rpl22l1*, *Slc2a1*, *Slc16a3*, *Hist1h2ac*, *Fam162a*, *Nbeal2*, *Ppia*, *Asf1b*, *Mcm3*, *Mcm7*, *Mcm10*, *Ccne2*, *Ccna2*, *Fam64a*, *Cdca5*, *Cdc2*a, *Cdc20*, *Tuba1b*, *Myl4*, *Nusap1*, *Sash1*, *Mgarp*, *Eef1a1*, *Pkm*2, *Pfkfb3*, *Pdk1*, *Pgk1*, *Pfkl*, *Aldoa*, *Aldoc*, *Pygl*, *Ldha*, *Pgam1*, *Tpi1*, *Gapdh*, *Aurka*, *Aurkb*, *Acta1*, *Mycn*, *Btg2*, *Ccng1*, *9030617O03Rik*, and *Myh6* were purchased from GE Healthcare (siGENOME, SMARTpool; Dharmacon-GE Healthcare, CA, USA). The siRNAs for *Hif-1α* (#s67530), *Mif* (#s233127), *Fam64a* (#s99452), *Tuba1b* (#s75582), *Hist1h2ao* plus *Hist1h2af* (#s234498), *Hist2h2ac* (#s232707), *Hist1h2ak* (#s234536), *Cdh1* (#s80429), and *Gm5069* (#n256492) were purchased from Thermo-Fisher (Silencer Select; Ambion-Thermo-Fisher). The siRNA for *GFP* was purchased from Thermo-Fisher (Silencer; Ambion-Thermo-Fisher). The siRNA for *Egln3* was purchased from TaKaRa Bio (Japan). Corresponding negative control siRNAs were also used in each experiment.

Immunofluorescence.

For tissue analyses, frozen heart sections embedded in OCT compound (Tissue-Tek®; Sakura, UAE) were cut into 8 µm sections with a cryostat (CM3050S; Leica Biosystems, Germany), permeabilized with 0.1% Triton X-100, blocked with Blocking-One (Nacalai Tesque, Japan), and double-labeled with primary antibodies for sarcomeric α-actinin (Sigma-Aldrich) and Fam64a. The latter antibody was raised against a synthetic peptide corresponding to residues 102-114 of mouse Fam64a (CQSGTKWLMETQV). Samples were then labeled with fluorochrome-conjugated secondary antibodies (Thermo-Fisher) as described[4,5]. When necessary, 4', 6-diamidino-2-phenylindole (DAPI) staining for DNA or phalloidin staining for F-actin filaments was also performed. The same protocol was applied for cultured cells, except that fixation with 4% paraformaldehyde was first performed before permeabilization. Primary antibodies used were against Ki67 (clone SP6; Abcam), phospho-histone H3 at Ser-10 (EMD Millipore, MA, USA), sarcomeric α-actinin (Sigma-Aldrich), and Fam64a (Bioss Antibody, GA, USA). Another Fam64a antibody, which was raised as stated above, was also used. When using mouse-derived antibodies, the Mouse on Mouse (M.O.M.™) Basic Kit (Vector, CA, USA) was used. Cells or sections covered with fluorescence mounting medium (DAKO, CA, USA) were examined using a confocal microscope (Fluoview™ FV1000; Olympus) mounted on an Olympus IX81 inverted microscope.

Immunoblotting.

Hearts were collected from mice and snap frozen in liquid nitrogen. Minced tissues were homogenized using a Kinematica™ Polytron™ homogenizer (PT1600E; Fisher Scientific, MA, USA) in lysis buffer (10 mmol/L Tris-HCl, pH 7.5, 150 mmol/L NaCl, 0.5 mmol/L EDTA, 10 mmol/L NaF, and 0.5% Triton X-100), RIPA buffer (Thermo-Fisher), or M-PER buffer (Thermo-Fisher) in the presence of protease and phosphatase inhibitor cocktail (Thermo-Fisher or Roche, Basel, CH). For cultured CMs, harvested cell pellets were lysed in the same buffer and processed as was done for heart tissues. Lysates were centrifuged at 14,000×*g* and supernatants were used as the whole protein extract. In some samples, nuclear extracts were subsequently obtained with nuclear extraction buffer (20

mmol/L Tris-HCl, pH 7.5, 20% glycerol, 0.5 mol/L NaCl, 1.5 mmol/L $MgCl_2$, 0.1% Triton X-100, and 1 mmol/L DTT). Nuclear fractions were suitable for the detection of Hif-1α. After quantifying total protein concentrations by the bicinchoninic acid (BCA) method (Thermo-Fisher), equal amount of proteins were separated by sodium dodecyl sulphate polyacrylamide gel electrophoresis (SDS-PAGE) (Mini-PROTEAN® TGX; Bio-Rad, CA, USA), and transferred onto polyvinylidene difluoride (PVDF) membranes (GE Healthcare). After blocking the membranes with BLOCK-ACE (DS Pharma, Japan) with 0.5% BSA or 5% nonfat milk, membranes were probed with primary antibodies, followed by secondary horseradish peroxidase (HRP)-conjugated IgG (GE Healthcare), and finally visualized by enhanced chemiluminescence (Western Lightning ECL-Pro; PerkinElmer, OH, USA) using a LAS4000mini luminescent image analyzer (GE Healthcare). Densitometry analysis was performed using Image J software. Primary antibodies used were: Rb (clone IF8; Santa Cruz), p53 (clone Pab 246; Santa Cruz or clone Pab 240; Abcam), mTOR (clone 7C10; Cell Signaling, MA, USA), phospho-mTOR at Ser-2448 (clone D9C2; Cell Signaling), AMPKα (clone 23A3; Cell Signaling), phospho-AMPKα at Thr-172 (clone 40H9; Cell Signaling), Hif-1α (LifeSpan BioSciences, WA, USA), Mif (clone EPR12463; Abcam), β-tubulin (clone 9F3; Cell Signaling), and GFP (clone B-2; Santa Cruz).

Quantitative real-time polymerase chain reaction (qPCR).

Hearts were collected from mice, cut into small pieces, and immediately immersed in RNAlater® RNA Stabilization Reagent (QIAGEN). The stabilized tissues were homogenized with a Micro Smash™ homogenizer (MS-100R; Tomy, Japan), and total RNA was isolated using the RNeasy® Plus Mini Kit (QIAGEN), the RNeasy® Fibrous Tissue Mini Kit (QIAGEN), or the ISOGEN system (Nippon Gene, Japan). For cultured CMs, harvested cell pellets in appropriate buffer were processed as was done for heart tissues. After assessing RNA yield and quality using a NanoDrop spectrophotometer (ND-1000; Thermo-Fisher), RNA samples were reverse-transcribed with PrimeScrip RT Master Mix (TaKaRa Bio), and quantitative real-time PCR was performed using the TaqMan® Fast Advanced Master Mix in a StepOnePlus™ real-time PCR system (Applied Biosystems).

TaqMan® gene expression assays used were for *PolK* (#Mm00449572_m1), *Ccng1* (#Mm00438084_m1), *Pierce1* (#Mm01332441_m1), *Pmaip1* (#Mm00451763_m1), *Ccna2* (#Mm00438063_m1), *Ccne2* (#Mm00438077_m1), *Egln1* (#Mm00459770_m1), *Egln2* (#Mm00519067_m1), *Egln3* (#Mm00472200_m1), *Mif* (#Mm01611157_gH), *Hif-1α* (#Mm00468869_m1), *Aurkb* (#Mm01718146_g1), *Cdca5* (#Mm01233533_m1), *Nusap1* (#Mm00505601_m1), *Myl4* (#Mm00440377_m1), *Tuba1b* (#Mm02030931_s1), *Tubb5* (#Mm00495806_g1), and *Fam64a* (#Mm01245821_g1). Quantification of each mRNA was carried out with *Actb* (#Mm00607939_s1), *Rn18S* (#Mm03928990_g1), *18S* (#Hs99999901_s1), or *Ubc* (#Mm01198158_m1) as reference genes using the $\Delta\Delta C_T$ method.

Chromatin immunoprecipitation (ChIP) assay.

ChIP assays were performed using EpiScope ChIP Kit (Takara Bio) as per the manufacturer's protocol using HEK293T/17 cells or fetal mouse heart tissues. HEK293T/17 cells were crosslinked with 1% formaldehyde for 5 min at room temperature. Fetal mouse hearts were crosslinked with 1–1.8% formaldehyde for 2–3h at room temperature and homogenized. Samples were then sonicated with Bioruptor (Cosmo Bio, Japan) and subjected to IP using antibodies against HIF-1α (Santa Cruz), HIF-2α (Novus Biologicals, CO, USA) or normal mouse IgG (Santa Cruz). Purified DNA was quantified using SYBR Green real-time PCR system (Takara Bio). Six primers for humans and five primers for mice were designed to include putative Hif-1α binding sites (5'-GCGTG-3') (Supplementary Fig 4d for human promoter sequence). Fold enrichment was calculated based on Ct as $2^{-\Delta(\Delta Ct)}$, where $\Delta Ct = Ct_{IP} - Ct_{input}$ and $\Delta(\Delta Ct) = \Delta Ct_{Hif\text{-}1\alpha \text{ or } Hif\text{-}2\alpha} - \Delta Ct_{IgG}$. In some experiments, cells were cultured in low O_2 conditions or treated with PHD inhibitor DMOG to stabilize HIF-1α protein.

Luciferase reporter assay.

HEK293T/17 cells or fCMs (E16–17) were plated onto 96 well plates, transfected with LightSwitch™ Promoter Reporter GoClone™, which contains human *FAM64A* promoter sequence cloned upstream of the RenSP luciferase gene in the pLightSwitch_Prom reporter vector (SwitchGear Genomics, CA,

USA) using Lipofectamine® 2000. For Hif-1α stabilization, cells were treated with PHD inhibitor DMOG at 500uM for 24h in high O_2 culture conditions. For Hif-1α silencing, cells were transfected with siRNA targeting Hif-1α using Lipofectamine® RNAiMAX in low O_2 culture conditions. Luciferase activity was measured using LightSwitch™ luciferase assay reagent (SwitchGear Genomics) as per the manufacturer's protocol. Luciferase activities of housekeeping gene ribosomal protein L10 (*RPL10*) and empty vector were used as positive reference and negative background control, respectively.

Statistics.

All data were expressed as mean plus or minus standard error of the mean (SEM). For comparisons between two groups, Student's two-tailed paired *t*-test was used to determine statistical significance. For comparisons among multiple groups, one-way analysis of variance (ANOVA) was used with Bonferroni's post hoc test. $P<0.05$ was considered statistically significant. Significance levels were indicated as $^{*}P<0.05$, $^{**}P<0.01$, and $^{***}P<0.001$.

Supplementary References

1. Katanosaka Y. *et al*. TRPV2 is critical for the maintenance of cardiac structure and function in mice. *Nat Commun*. **5,** 3932; 10.1038/ncomms4932 (2014).

2. Hashimoto K. *et al*. Monocyte trans-endothelial migration augments subsequent transmigratory activity with increased PECAM-1 and decreased VE-cadherin at endothelial junctions. *Int J Cardiol.* **149,** 232-239 (2011).

3. Tani H., Nishijima M., Ushijima H., Miyamura T. & Matsuura Y. Characterization of cell-surface determinants important for baculovirus infection. *Virology.* **279,** 343-353 (2001).

4. Hashimoto K., Kataoka N., Nakamura E., Tsujioka K. & Kajiya F. Oxidized LDL specifically promotes the initiation of monocyte invasion during transendothelial migration with upregulated PECAM-1 and downregulated VE-cadherin on endothelial junctions. *Atherosclerosis.* **194,** e9-e17

(2007).

5. Ujihara Y. *et al*. Induced NCX1 overexpression attenuates pressure overload-induced pathological cardiac remodelling. *Cardiovasc Res.* **111,** 348-361 (2016).

Fam64a is a novel cell cycle promoter of hypoxic fetal cardiomyocytes in mice.

*Ken Hashimoto, Aya Kodama, Takeshi Honda, Akira Hanashima, Yoshihiro Ujihara, Takashi Murayama, Shin-ichiro Nishimatsu, Satoshi Mohri

Legend for Supplementary Movies 1-6

Supplementary Movies 1, 2 Representative time-lapse recordings of cell division dynamics of fCMs under low O_2 isolation/culture protocol. Complete mitosis, cytokinesis, and the generation of new daughter cells were clearly visible at the single-cell level, providing intuitive evidence for the sequence of fCM division events. Before division, cells rounded up, as is common with proliferating cells (particularly evident in Supplementary Movie 1). Supplementary Movie 2 corresponds to the time series shown in Fig 2b. Supplementary Movie 1 is an additional time series not shown in the figure. The number on each panel indicates the time in minutes elapsed from the beginning, which is adjusted to the first panel in Fig 2b. Thus the earlier time point in full movies is expressed as a negative value. Scale bar=30μm.

Supplementary Movie 3 Representative time-lapse recordings of α-actinin-expressing fCMs undergoing cell division. Complete mitosis, cytokinesis, and the generation of new daughter cells were clearly visible at the single-cell level. This movie corresponds to the time series shown in Fig 2d and is provided as two separate sub-movies (-1: α-actinin-mCherry channel, and -2: phase contrast channel). The number on each panel indicates the time in (minutes:seconds) elapsed from the beginning, which is adjusted to the first panel in Fig 2d. Thus the earlier time point in full movies is expressed as a negative value.

Supplementary Movies 4–6 Representative time-lapse recordings of cell division dynamics of fCMs and fibroblasts transduced with WT Fam64a-GFP baculovirus. Complete mitosis, cytokinesis, and the generation of new daughter cells were clearly visible at the single-cell level, providing intuitive evidence for Fam64a molecular dynamics during division. While the rapid disappearance of Fam64a-GFP signal was observed before anaphase in fCMs (Supplementary Movies 4, 5), it occurred later at the completion of mitosis in fibroblasts (Supplementary Movie 6). Supplementary Movies 4 and 6 correspond to the time series shown in Fig 6a and 6b, respectively. These movies are provided as two separate sub-movies (-1: merged images with phase

contrast images, and -2: Fam64a-GFP channel alone). Supplementary Movie 5 is an additional time series of fCMs not shown in the figure (merged image only). The number on each panel indicates the time in (hours:minutes) elapsed from the beginning, which is adjusted to the first panel in Fig 6a and 6b. Thus the earlier time point in full movies is expressed as a negative value. Scale bar=30μm.
